# Personalized Prediction of Long-Term Renal Function Prognosis Following Nephrectomy Using Interpretable Machine Learning Algorithms: Case-Control Study

**DOI:** 10.2196/52837

**Published:** 2024-09-20

**Authors:** Lingyu Xu, Chenyu Li, Shuang Gao, Long Zhao, Chen Guan, Xuefei Shen, Zhihui Zhu, Cheng Guo, Liwei Zhang, Chengyu Yang, Quandong Bu, Bin Zhou, Yan Xu

**Affiliations:** 1 Department of Nephrology, The Affiliated Hospital of Qingdao University Qingdao China; 2 Medizinische Klinik und Poliklinik IV Klinikum der Universität Munich Germany; 3 Ocean University of China, Qingdao, CN Qingdao China; 4 Center of Structural Heart Disease Beijing Anzhen Hospital Capital Medical University Beijing China; 5 Allianz Technology Allianz Munich Germany; 6 Institute of Diabetes and Regeneration Research, Helmholtz Diabetes Center Helmholtz Center Munich Neuherberg Germany

**Keywords:** nephrectomy, acute kidney injury, acute kidney disease, chronic kidney disease, machine learning

## Abstract

**Background:**

Acute kidney injury (AKI) is a common adverse outcome following nephrectomy. The progression from AKI to acute kidney disease (AKD) and subsequently to chronic kidney disease (CKD) remains a concern; yet, the predictive mechanisms for these transitions are not fully understood. Interpretable machine learning (ML) models offer insights into how clinical features influence long-term renal function outcomes after nephrectomy, providing a more precise framework for identifying patients at risk and supporting improved clinical decision-making processes.

**Objective:**

This study aimed to (1) evaluate postnephrectomy rates of AKI, AKD, and CKD, analyzing long-term renal outcomes along different trajectories; (2) interpret AKD and CKD models using Shapley Additive Explanations values and Local Interpretable Model-Agnostic Explanations algorithm; and (3) develop a web-based tool for estimating AKD or CKD risk after nephrectomy.

**Methods:**

We conducted a retrospective cohort study involving patients who underwent nephrectomy between July 2012 and June 2019. Patient data were randomly split into training, validation, and test sets, maintaining a ratio of 76.5:8.5:15. Eight ML algorithms were used to construct predictive models for postoperative AKD and CKD. The performance of the best-performing models was assessed using various metrics. We used various Shapley Additive Explanations plots and Local Interpretable Model-Agnostic Explanations bar plots to interpret the model and generated directed acyclic graphs to explore the potential causal relationships between features. Additionally, we developed a web-based prediction tool using the top 10 features for AKD prediction and the top 5 features for CKD prediction.

**Results:**

The study cohort comprised 1559 patients. Incidence rates for AKI, AKD, and CKD were 21.7% (n=330), 15.3% (n=238), and 10.6% (n=165), respectively. Among the evaluated ML models, the Light Gradient-Boosting Machine (LightGBM) model demonstrated superior performance, with an area under the receiver operating characteristic curve of 0.97 for AKD prediction and 0.96 for CKD prediction. Performance metrics and plots highlighted the model’s competence in discrimination, calibration, and clinical applicability. Operative duration, hemoglobin, blood loss, urine protein, and hematocrit were identified as the top 5 features associated with predicted AKD. Baseline estimated glomerular filtration rate, pathology, trajectories of renal function, age, and total bilirubin were the top 5 features associated with predicted CKD. Additionally, we developed a web application using the LightGBM model to estimate AKD and CKD risks.

**Conclusions:**

An interpretable ML model effectively elucidated its decision-making process in identifying patients at risk of AKD and CKD following nephrectomy by enumerating critical features. The web-based calculator, found on the LightGBM model, can assist in formulating more personalized and evidence-based clinical strategies.

## Introduction

Renal tumors rank as the second most prevalent neoplasms in urology, succeeding bladder cancer, and their annual incidence is on the rise [1,2]. Nephrectomy remains the preferred therapeutic modality for localized renal tumors [3], and patients who are eligible for nephrectomy generally favor longer life span [4]. Nevertheless, a decline in kidney function frequently ensues following nephrectomy. It has been proven that the nephron reduction stemming from radical nephrectomy (RN) or partial nephrectomy (PN) can result in postoperative acute kidney injury (AKI), subsequently heightening the risk of chronic kidney disease (CKD) and mortality [5,6]. Therefore, it is crucial to discern the predicted risk factors associated with renal function decline and precisely forecast postoperative renal impairment, enabling timely intervention.

AKI and CKD are not 2 separate clinical syndromes but often manifest as a continuum of disease [7]. The 16th Acute Disease Quality Initiative meeting has defined acute kidney disease (AKD) as the occurrence of acute or subacute damage or loss of kidney function for a duration of 7 to 90 days after the onset of an AKI-initiating event [8]. Within the AKD time frame, interventions like patient education, medication adjustments, and regular follow-up can be initiated, potentially leading to disease reversal [9]. According to AKD definition, renal recovery is classified into 3 primary groups: transient AKI, subacute AKD, and persistent AKI [8].

Recent, noteworthy strides in machine learning (ML) have given rise to remarkable breakthroughs, encompassing fields like autonomous driving, recommending products, and surpassing human expertise in intricate games such as chess [10-12]. These advancements have increasingly impacted the health care domain, particularly in clinical decision support systems, aiding in clinical decision-making, forecasting disease progression, and enhancing the distribution of medical resources [13,14]. ML offers significant advantages in clinical decision-making by analyzing large datasets, facilitating high-throughput and real-time predictions, and identifying complex patterns. However, considering the challenges related to decision-making transparency, individual patient variability, and ethical concerns, ML should be considered a complementary tool to enhance physicians’ diagnostic capabilities rather than substituting their expertise. One of the prominent challenges faced by ML in the health care domain is the enigma referred to as the “black-box phenomenon,” indicating the deficiency in interpretability experienced by both patients and health care providers [15,16]. The absence of interpretability in predictive models can erode trust in these models, particularly in health care, where numerous decisions directly involve matters of life and death. Recent advancements, however, have introduced algorithms that effectively extract crucial variables and elucidate model decisions [17].

Currently, there is limited research on the risk prediction of AKD following nephrectomy, and the impact of postnephrectomy AKD on CKD remains unclear. Additionally, there is a lack of interpretable ML models and web-based prediction tools for both AKD and CKD. Therefore, this study aimed to achieve the following objectives: (1) assess the postoperative occurrence rates of AKI, AKD, and CKD in patients who underwent nephrectomy; (2) contrast the long-term renal prognosis across AKI recover, subacute AKD, and patients with AKD and AKI; (3) formulate risk prediction models for both AKD and CKD through the use of diverse ML algorithms; (4) determine the optimal models, evaluate their predictive efficacy, and explain via Shapley Additive Explanations (SHAP) values and Local Interpretable Model-Agnostic Explanations (LIME) algorithms; (5) use directed acyclic graphs (DAGs) to explore potential associations and causal pathways between features; and (6) devise an easily accessible web-based prediction tool tailored to estimating the likelihood of AKD and CKD after nephrectomy. We hypothesized that patients with acute or subacute renal impairment are more susceptible to CKD progression compared to those with normal renal function. Furthermore, we expected significant differences in the development of CKD among patients recovering from AKI, those with subacute AKD, and those experiencing AKD with AKI.

## Methods

### Study Design

We conducted a retrospective review of medical records for 2637 patients who underwent nephrectomy between July 2012 and June 2019. Ultimately, the study included 1559 eligible patients. The patient data were sourced from a prominent tertiary hospital known for its comprehensive services and ranked among the top 60 nationwide in terms of overall performance. Patients were followed up for a duration ranging from 3.0 to 62.8 months until December 2019, with the primary focus being on the development of CKD as a long-term outcome. The patient data were randomly stratified into training, validation, and test sets, using Python’s stratified random sampling method, maintaining a ratio of 76.5:8.5:15. Internal validation was performed through 10-fold cross-validation, involving the partitioning of the training and validation sets into 10 subsets. A majority of 9 of these subsets were used for model training, and the remaining 1 was dedicated to model evaluation. The exclusion criteria for this study encompassed the following characteristics: (1) patients younger than 18 years of age or with hospitalization duration <24 hours, (2) patients with inadequate serum creatinine (Scr) monitoring interval, (3) patients with anatomical kidney malformations, (4) patients undergoing renal cyst unroofing or donor nephrectomies, (5) patients with pre-existing CKD or undergoing dialysis prior to nephrectomy, and (6) patients lacking essential features such as Scr.

### Ethical Considerations

This study received approval from the Ethics Committee of the Affiliated Hospital of Qingdao University (approval QDFY WZ 2018-9-13). Informed consent was waived due to the retrospective nature of the data and the large number of patients involved, making it impractical to seek consent from each patient. All data were deidentified. No compensation was provided to the participants as the study did not involve direct participant interaction.

### Data Collection

Clinical and demographic data were extracted through the application of natural language processing and parsing methods on structured information within the electronic health record. Preoperative complete blood counts, coagulation markers, blood chemistry analyses, urine tests, and echocardiography were performed within 3 days of admission. Comorbidities were defined based on the *International Statistical Classification of Diseases, Tenth Revision*. Comprehensive data on concomitant medications were meticulously collected, with particular attention to instances where these medications were administered prior to the occurrence of kidney injury. The surgical details encompass the surgical approach (laparotomy, laparoscopy, and da Vinci surgery), procedure type (RN and PN), duration of the surgery, pathological findings, maximum excision diameter, and blood loss.

### Outcome Definitions

The primary outcome of our study was postoperative AKI. The secondary outcomes were AKD and CKD. AKI was defined as an increase in Scr to ≥0.3 mg/dL within 48 hours or ≥1.5 times the baseline value within 7 days, following the 2012 Kidney Disease Improving Global Outcomes guideline [18]. According to the 2017 Acute Disease Quality Initiative, AKD was defined as persistent renal damage or renal dysfunction for a duration of 7 to 90 days after exposure to an AKI initiating event [8]. CKD was defined as abnormalities of kidney structure or function for at least 3 months [19]. Based on the diagnostic criteria for AKI and AKD, patients exhibited three distinct trajectories of renal function following kidney injury: (1) AKI recover, if Scr returned to baseline value within 7 days (AKI without AKD); (2) AKD with AKI, if stage 1 or greater AKI persisted for ≥7 days after an AKI initiating event (continuous AKI progressing to AKD); and (3) subacute AKD, if Scr levels increased slowly but lasted more than 7 days (AKD without AKI). The final classification consisted of four categories: (1) no kidney disease (NKD), (2) AKI recover, (3) AKD with AKI, and (4) subacute AKD.

Baseline Scr was defined as the most recent Scr level measured before nephrectomy. The diagnosis time of AKI, AKD, and CKD was established when patients first met the respective diagnostic criteria. All patients underwent at least 3 Scr tests, including 2 during hospitalization and 1 at the first follow-up. If elevated Scr levels did not return to baseline, additional tests were conducted once a week during hospitalization or at the next follow-up. The estimated glomerular filtration rate (eGFR) was calculated by using the Chronic Kidney Disease Epidemiology Collaboration creatinine formula [20].

### Model Development and Interpretation

The Light Gradient-Boosting Machine (LightGBM) algorithm was used to construct predictive models. LightGBM, a tree-based gradient-boosting framework, adeptly manages high-dimensional and extensive datasets [21]. By integrating gradient-based 1-side sampling and exclusive feature bundling, LightGBM effectively mitigates overfitting and notably outperforms the computational speed and memory use of Extreme Gradient-Boosting and stochastic gradient-boosting techniques [22]. In our comparative analysis, we trained various ML models, including LightGBM, Gradient-Boosting Machine, k-nearest neighbors, multilayer perceptron, logistic regression (LR), naive Bayes, random forest (RF), and support vector machine, using the same dataset and applying consistent imputation and scaling techniques. We initially used the default hyperparameters of each ML algorithm to establish our models. Subsequently, we conducted manual parameter tuning by grid search to optimize the performance. The process of parameter optimization was facilitated through 10-fold cross-validation, aiding in the identification of the most suitable hyperparameter configurations [23].

For discerning significant features that influenced the algorithm and ensuring the appropriateness of the optimal model, we used SHAP and LIME to interpret the model from both global and instance-based perspectives. SHAP values, rooted in the Shapley value from coalitional game theory, quantify the influence of each feature variable on the target outcome, elucidating the derivation of a sample’s predicted result [24]. LIME uses local surrogate models for explaining individual predictions. Its core method perturbs an input instance to generate interpretable samples, upon which a linear model approximates the complex model’s decision-making process near the instance [25]. The SHAP summary plots exhibit the relative significance of individual features in predictions, along with their corresponding positive or negative impact directions. The SHAP interaction plots reveal the interactions among multiple features and illustrate how their combined influence impacts model predictions. We separately used the top 10 features from the AKD and CKD models and created SHAP dependence plots through pairwise combinations to elucidate the influence of individual features on the model’s predictions and the correlations among them. Additionally, we highlighted features with significant correlations in Figure S5 in Multimedia Appendix 1. The SHAP force plots and LIME bar plots were used to clarify individualized forecasts, demonstrating each feature’s contribution to the prediction of individual samples. Finally, we used AKD and CKD as outcomes and applied the PC algorithm to construct DAGs, facilitating the exploration of potential associations and causal pathways among the top 20 features [26,27].

### Web-Based Prediction Tool

A web-based calculator for predicting AKD and CKD among those patients was developed using the “Streamlit” application in terms of the optimal model. Streamlit, an open-source Python framework, aids developers in swiftly constructing web-based and responsive applications [28]. To improve the user-friendliness of the web calculator, this study implemented 2 panels: one for inputting model parameters and acquiring AKD or CKD probabilities and another for providing a model introduction.

### Statistical Analysis

Features with a missing proportion exceeding 15% (n=234) are removed, while those with missing proportions less than 15% (n=234) are imputed using an RF model. Using LR to calculate the required sample size with CKD as the outcome, we determined that a minimum of 1171 patients is necessary to attain a statistical power of 90% for detecting an effect size of 0.10 at a 2-side α=.05. Categorical features were presented using frequencies and percentages, while continuous features were presented as mean (SD) or median (IQR). Comparative analyses were performed to assess patient characteristics between individuals with and without CKD as well as among various trajectories of renal function postkidney injury. Quantile-quantile plots were generated to visually inspect the distribution patterns of continuous features. The independent 2-tailed *t* test was used for normally distributed continuous features, the Mann-Whitney *U* test for nonnormally distributed continuous features, and the Pearson chi-square test for categorical features. We used a weight rebalancing technique to adjust the weights of both the majority and minority classes in the training dataset [29]. The validation dataset underwent balancing, whereas the test datasets remained unaltered to assess model performance with representative data. The scikit-learn Python library (Python Software Foundation) includes a built-in parameter called “class weight” or “weights” for LR, RF, LightGBM, support vector machine, and k-nearest neighbors. For AKD, the class weight was set to 3.3; and for non-AKD cases, it was set to 0.6. Similarly, the class weight for CKD was set to 10.0; and for non-CKD cases, it was set to 0.5. In the case of the naive Bayes classifier, we established a prior probability of .50 for each class to achieve group balance, and we adjusted class weights in the multilayer perceptron classifier by modifying the loss function’s weights. The area under the receiver operating characteristic curve (AUROC) was used for optimal model selection. The model underwent evaluation through graphical techniques, encompassing the receiver operating characteristic curve and decision curve analysis, in addition to quantitative metrics such as AUROC, average precision, precision, recall, accuracy, *F*_1_-score, Brier score loss, and Matthew correlation coefficient. A *P* value of less than .05 was considered as significant (2-tailed). Python programming language (version 3.9.13 and integrated development environment Visual Studio Code 1.81.1) was applied to our analysis.

## Results

### Study Cohort

**Figure 1:**
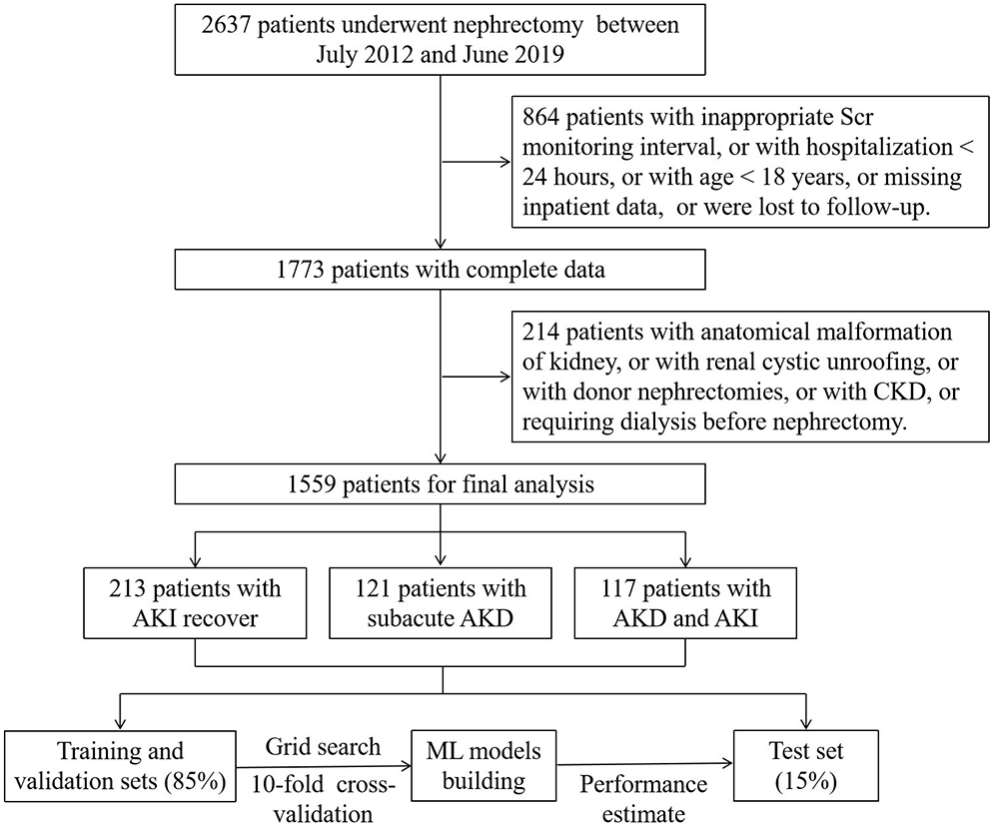
Flow diagram of patients’ enrollment. AKD: acute kidney disease; AKI: acute kidney injury; CKD: chronic kidney disease; ML: machine learning; Scr: serum creatinine.

The entire study process is illustrated in Figure 1. Among the participants, 1131 (72.6%) underwent RN, and 1152 (73.9%) underwent laparotomy. The incidence rates of AKI, AKD, and CKD were 21.7% (330/1559), 15.3% (238/1559), and 10.6% (165/1559), respectively. In total, there were 451 (28.9%) patients who developed acute or subacute kidney dysfunction (AKI or AKD criterion), with 117 (7.5%) meeting both AKI and AKD criteria, 121 (7.8%) developed subacute AKD, and 213 (13.7%) experienced recovery from AKI. The quantile-quantile plots show that features including blood loss, Scr, and operative duration exhibit skewed distributions, potentially due to the distinct condition of nephrectomy patients (Figure S1 in Multimedia Appendix 1). Increased CKD rates were observed in older patients (mean age 69, SD 9.6 vs mean age 58, SD 12.3 years), male patients (n=118, 12.9% vs n=47, 7.3% in female patients), those who underwent RN (n=143, 12.6% vs n=22, 5.1% in PN), AKD with AKI (n=42, 35.9% vs n=32, 26.4% in subacute AKD, n=24, 11.3% in AKI recovery, and n=67, 6% in NKD), and individuals with 1 or more chronic complications such as hypertension, diabetes mellitus, and coronary heart disease. The demographic and clinical characteristics of the patient cohort, both within different groups and as a whole, are detailed in Table 1 and Table S1 in Multimedia Appendix 2.

**Table 1:** Baseline characteristics of patients with and without CKD^a^.

Features		Total (N=1559)	CKD-free (n=1394)	CKD (n=165)	*P* value
Age (years), mean (SD)		59.1 (12.5)	58 (12.3)	68.5 (9.6)	<.001
Male, n (%)		914 (58.6)	796 (57.1)	118 (71.5)	<.001
BMI (kg/m^2^), mean (SD)		25 (3.4)	24.9 (3.4)	25.7 (3.8)	.01
Systolic blood pressure (mm Hg), mean (SD)		131.9 (18.5)	130.9 (18.2)	139.8 (19.3)	<.001
Smokers, n (%)		570 (36.6)	490 (35.1)	80 (48.5)	<.001
Drinkers, n (%)		464 (29.8)	404 (29)	60 (36.4)	.06
Fever, n (%)		56 (3.6)	52 (3.7)	4 (2.4)	.53
**Procedure, n (%)**					
	Radical nephrectomy	1131 (72.5)	988 (70.9)	143 (86.7)	<.001
	Partial nephrectomy	428 (27.4)	406 (29.1)	22 (13.3)	—^b^
**Approach, n (%)**					
	Laparotomy	1152 (73.9)	1038 (74.5)	114 (69.1)	.29
	Laparoscopy	316 (20.3)	275 (19.7)	41 (24.8)	—
	da Vinci surgery	91 (5.8)	81 (5.8)	10 (6.1)	—
**Pathology, n (%)**		1973 (126.6)	1721 (123.5)	252 (152.7)	—
	Benign	771 (73. 9)	673 (48.3)	98 (59.4)	<.001
	Malignant (nonclear)	431 (20.3)	375 (26.9)	56 (33.9)	—
	Clear cell	357 (5.8)	346 (24.8)	11 (6.7)	—
Blood loss (mL), median (IQR)		50 (20-150)	50 (20-150)	100 (50-200)	<.001
Excision diameter (cm), median (IQR)		11 (7-13)	12 (10-14)	11 (6-13)	<.001
Operative duration (hours), median (IQR)		2.5 (2-3)	2.8 (2.3-3.3)	2.4 (2-3)	<.001
**Laboratory tests**					
	White blood cell (×10^9^/L), median (IQR)	6.1 (5.1-7.4)	6.6 (5.6-7.8)	6 (5-7.4)	<.001
	Red blood cell (×10^12^/L), mean (SD)	4.5 (0.6)	4.5 (0.6)	4.4 (0.6)	.01
	Platelet (×10^9^/L), median (IQR)	232 (193-278)	221 (190-262)	234 (193-280)	.02
	Hemoglobin (g/L), mean (SD)	133.9 (20.4)	134.3 (20.2)	130 (21.3)	.01
	Fibrinogen (g/L), median (IQR)	3 (2.6-3.6)	3.1 (2.7-3.7)	3 (2.5-3.6)	.01
	Serum creatinine (μmol/L), median (IQR)	85 (73-97)	102 (91-121)	83 (72-95)	<.001
	Blood urea nitrogen (mmol/L), median (IQR)	5.7 (4.7-6.8)	6.6 (5.6-7.9)	5.6 (4.6-6.7)	<.001
	Uric acid (μmol/L), mean (SD)	316.5 (89.2)	312 (87.8)	354.2 (92.5)	<.001
	Baseline estimated glomerular filtration rate (mL/min/1.73 m^2^), mean (SD)	79.3 (18.7)	81.8 (17.4)	57.6 (15)	<.001
	Alanine transaminase (U/L), median (IQR)	17 (13-24)	17 (13-22)	18 (13-24)	.19
	Aspartate transaminase (U/L), median (IQR)	17 (14-20)	16 (13-19)	17 (14-20)	.11
	Total bilirubin (μmol/L), median (IQR)	13.1 (10-17.7)	11.8 (9.4-15.5)	13.4 (10.1-17.9)	<.001
	Alkaline phosphatase (U/L), median (IQR)	69 (57-84)	68 (57-78)	69 (57-85)	.23
	Triglyceride (mmol/L), median (IQR)	1.1 (0.8-1.6)	1.2 (0.9-1.7)	1.1 (0.8-1.6)	.01
	Low-density lipoprotein cholesterol (mmol/L), mean (SD)	2.9 (0.8)	2.9 (0.8)	2.8 (0.8)	.40
	Albumin (g/L), mean (SD)	39.5 (5)	39.7 (5)	38.4 (5.1)	<.001
	Blood glucose (mmol/L), median (IQR)	5.1 (4.6-5.8)	5.2 (4.8-6.2)	5.1 (4.6-5.8)	.01
**Urinalysis, n (%)**					
	Protein	307 (19.7)	230 (16.5)	77 (46.7)	<.001
	Glucose	206 (13.2)	177 (12.7)	29 (17.6)	.10
	Hematuria	1002 (64.3)	857 (61.5)	145 (87.8)	<.001
**Echocardiography, median (IQR)**					
	Ejection fraction	63 (61-65)	63 (61-65)	63 (61-65)	.62
**Comorbidities, n (%)**					
	Diabetes mellitus	202 (13)	166 (11.9)	36 (21.8)	<.001
	Coronary heart disease	124 (7.9)	94 (6.7)	30 (18.2)	<.001
	Hypertension	488 (31.3)	396 (28.4)	92 (55.8)	<.001
	Obesity	302 (19.4)	255 (18.3)	47 (28.5)	<.001
**Medications, n (%)**					
	β-Blocker	630 (40.4)	557 (40)	73 (44.2)	.33
	ACEI or ARB^c^	163 (10.5)	129 (9.2)	34 (20.6)	<.001
	Calcium channel blocker	378 (24.2)	311 (22.3)	67 (40.6)	<.001
	Antibiotics	1042 (66.8)	918 (65.8)	124 (75.1)	.02
	Nonsteroidal anti-inflammatory drugs	416 (26.7)	367 (26.3)	49 (29.7)	.41
	Diuretics	435 (27.9)	373 (26.8)	62 (37.6)	<.001
**Trajectories of renal function, n (%)**					
	AKI^d^ recover	213 (13.6)	189 (13.6)	24 (14.5)	<.001
	Subacute AKD^e^	121 (7.8)	89 (6.4)	32 (19.4)	—
	AKD with AKI	117 (7.5)	75 (5.4)	42 (25.4)	—
**Outcome**					
	AKI, n (%)	330 (21.2)	264 (18.9)	66 (40)	<.001
	AKD, n (%)	238 (15.3)	164 (11.8)	74 (44.8)	<.001
	CKD, n (%)	165 (10.6)	0 (0)	165 (100)	<.001
	Length of stay, median (IQR)	11 (9-13)	11 (9-13)	11 (9-14)	<.001

^a^CKD: chronic kidney disease.

^b^Not available.

^c^ACEI or ARB: angiotensin-converting enzyme inhibitor or angiotensin receptor blocker.

^d^AKI: acute kidney injury.

^e^AKD: acute kidney disease.

### Model Performance

A comprehensive set of over 90 features was served as features for both AKD and CKD and were integrated into the ML models. Among the assessed ML models, the LightGBM model demonstrated superior performance (Figure S2 in Multimedia Appendix 1 and Tables S2 and S3 in Multimedia Appendix 2). In the test set, LightGBM achieved the highest AUROC of 0.97 for AKD and 0.96 for CKD prediction. The *F*_1_-scores, 0.75 for AKD and 0.70 for CKD, indicate a balanced trade-off between precision and recall. Additionally, Brier score loss was maintained at 0.05 for both AKD and CKD predictions, demonstrating the model’s impressive calibration. To create a user-friendly web-based calculator, we simplified the model by reducing the number of input features. The inclusion of the top 10 and top 5 features for the AKD and CKD models, respectively, negligibly affected the LightGBM model’s AUROC (achieving 0.94 vs 0.97 for AKD prediction and 0.94 vs 0.96 for CKD prediction, as detailed in Figure S2 in Multimedia Appendix 1 and Table 2). Notably, it outperformed all other ML algorithms in terms of AUROC (Tables S4 and S5 in Multimedia Appendix 2), while maintaining an optimal balance between precision, recall, and error rates (both false positives and negatives). Subsequently, we used the LightGBM model for result interpretation and the development of a web-based calculator. Comprehensive insights into performance metrics and visualizations are provided in Figures S2 and S3 in Multimedia Appendix 1, Table 2, and Tables S2-S5 in Multimedia Appendix 2.

**Table 2:** Performance of Light Gradient-Boosting Machine models in predicting AKD^a^ and CKD^b^ on the test set.

Outcome			AUROC^c^		Precision		Recall		Accuracy		False positive rate		False negative rate	*F*_1_-score		MCC^d^	BSL^e^
**AKD**																	
	Top 5 features	0.87		0.43		0.66		0.82		0.16		0.34		0.52	0.42		0.12
	Top 10 features	0.94		0.67		0.80		0.91		0.07		0.20		0.73	0.68		0.07
	Top 15 features	0.95		0.74		0.80		0.93		0.05		0.20		0.77	0.73		0.06
	Top 20 features	0.95		0.71		0.71		0.92		0.05		0.29		0.71	0.66		0.06
	All features	0.97		0.83		0.69		0.93		0.03		0.31		0.75	0.72		0.05
**CKD**																	
	Top 5 features	0.94		0.43		0.72		0.91		0.08		0.28		0.54	0.51		0.07
	Top 10 features	0.93		0.43		0.67		0.91		0.07		0.33		0.52	0.49		0.07
	Top 15 features	0.91		0.42		0.56		0.91		0.07		0.44		0.48	0.43		0.08
	Top 20 features	0.92		0.44		0.61		0.91		0.07		0.39		0.51	0.47		0.07
	All features	0.96		0.64		0.78		0.95		0.04		0.22		0.70	0.68		0.05

^a^AKD: acute kidney disease.

^b^CKD: chronic kidney disease.

^c^AUROC: area under the receiver operating characteristic curve.

^d^MCC: Matthew correlation coefficient.

^e^BSL: Brier score loss.

### Model Interpretation

**Figure 2:**
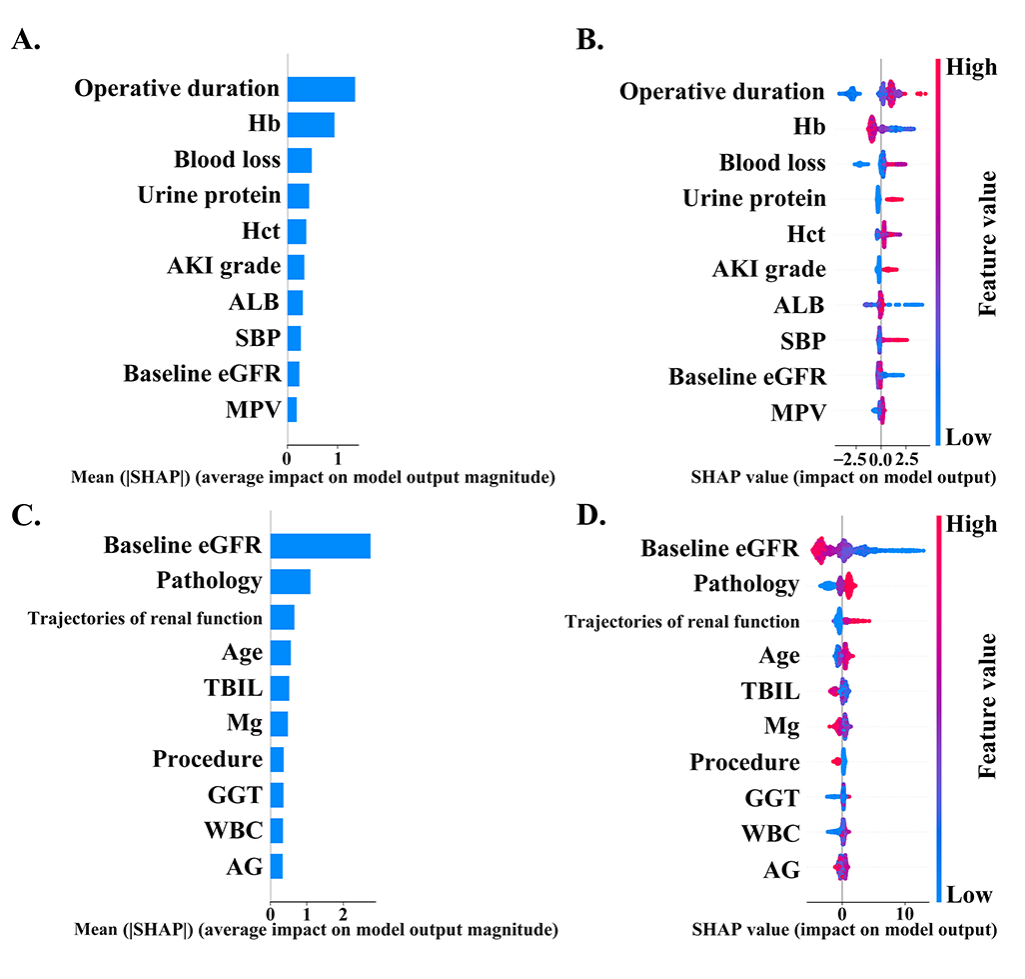
SHAP summary plots of the top 10 features in the Light Gradient Boosting Machine model for (A and B) AKD and (C and D) CKD prediction. (A) The ranking of feature importance within the AKD prediction model. Features with higher mean absolute SHAP values signify increased predictive influence. (B) Each dot represents the SHAP value of a specific feature for an individual, with red and blue indicating high and low feature values, respectively. On the x-axis, a positive or negative SHAP value signifies that the feature positively or negatively influenced the AKD prediction for the individual. (C) The ranking of feature importance within the CKD prediction model. (D) The distribution of the impacts of the top 10 features on the CKD model output. AG: albumin-globulin; AKD: acute kidney disease; AKI: acute kidney injury; ALB: albumin; CKD: chronic kidney disease; eGFR: estimated glomerular filtration rate; GGT: γ-glutamyl transferase; Hb: hemoglobin; Hct: hematocrit; MPV: mean platelet volume; SBP: systolic blood pressure; SHAP: Shapley Additive Explanations; TBIL: total bilirubin; WBC: white blood cell.

The SHAP summary plots of the LightGBM models are depicted in Figure 2. Operative duration, hemoglobin (Hb), blood loss, urine protein, and hematocrit were the top 5 features associated with predicted AKD. Baseline eGFR, pathology, trajectories of renal function, age, and total bilirubin were the top 5 features associated with predicted CKD. The SHAP interaction plots visually elucidate the interplays among the top 10 features in both the AKD and CKD models (Figure S4 in Multimedia Appendix 1). The SHAP dependence plots offer detailed insights into the correlations among the top 10 features, as depicted in Figures S6 and S7 in Multimedia Appendix 1, with representative examples showcased in Figure S5 in Multimedia Appendix 1. For instance, the influence of AKI grade on the probability of AKD varies across Hb levels. Among patients with lower Hb levels, higher AKI grades are associated with a significant increase in the risk of AKD. Conversely, this correlation is less pronounced in patients with higher Hb levels. For patients presenting with a baseline eGFR below 80, postoperative complications, such as AKD with AKI, subacute AKD, or AKI recover, markedly elevate the risk of developing CKD. This observation underscores the significance of encompassing factors like trajectories of renal function within a comprehensive clinical framework, particularly one that integrates a patient’s eGFR.

**Figure 3:**
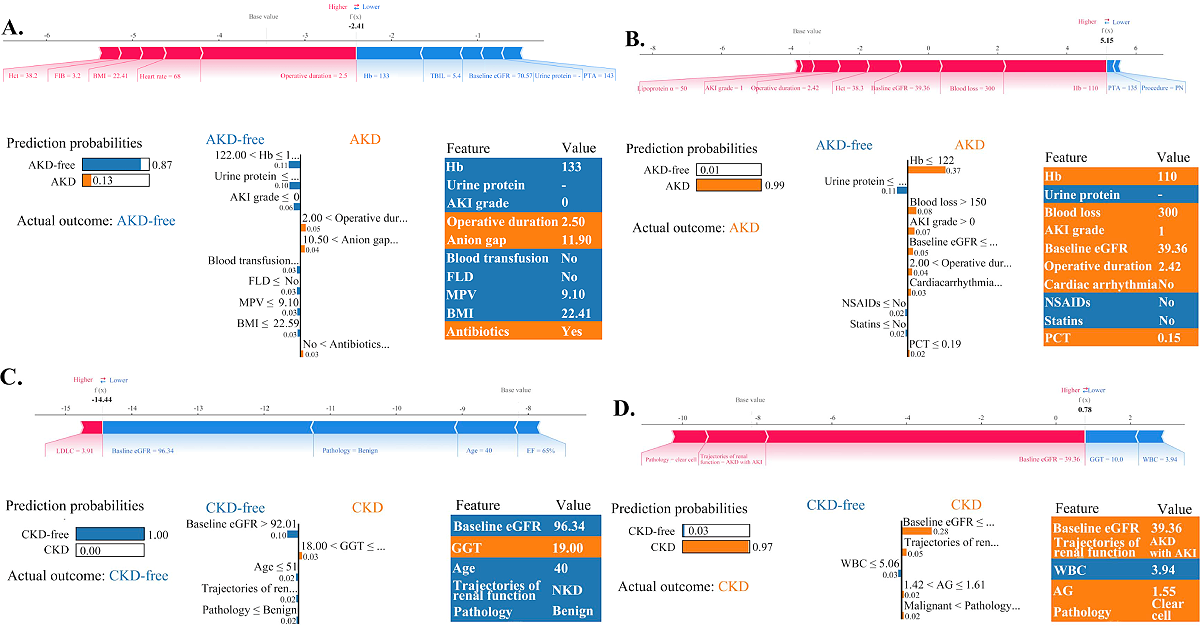
SHAP force plots and LIME bar plots for explaining individual predictions for (A and B) AKD and (C and D) CKD. (A) The SHAP force plot (upper section) and the LIME bar plot (lower section) are used to illustrate a case where both actual and predicted outcomes indicate AKD-free status. The SHAP force plot outlines the top 10 features for the prediction, where red feature values positively impact the AKD outcome, while blue values have a negative impact. The importance of each feature is reflected by the length of its corresponding arrow, with longer arrows highlighting more significant influences. In the LIME bar plot, the left section shows an 87% predicted probability of the patient being AKD-free. The central section lists the top 10 features for predicting AKD-free or AKD status, with the length of each bar indicating its importance. Blue bars indicate positive influences, whereas yellow bars signify negative impacts. The right panel presents the specific values at which these top 10 features have the most substantial impact on the AKD-free or AKD prediction. (B) The SHAP force plot and the LIME bar plot, emphasizing the top 10 features, depict a case where both actual and predicted outcomes align with AKD status. (C) The SHAP force plot and the LIME bar plot, emphasizing the top 5 features, depict a case where both actual and predicted outcomes align with CKD-free status. (D) The SHAP force plot and the LIME bar plot, emphasizing the top 5 features, depict a case where both actual and predicted outcomes align with CKD status. AG: albumin-globulin; AKD: acute kidney disease; AKI: acute kidney injury; ALB: albumin; CKD: chronic kidney disease; EF: ejection fraction; eGFR: estimated glomerular filtration rate; FIB: fibrinogen; FLD: fatty liver disease; GGT: γ-glutamyl transferase; Hb: hemoglobin; Hct: hematocrit; LDLC: low-density lipoprotein cholesterol; LIME: Local Interpretable Model-Agnostic Explanations; MPV: mean platelet volume; NKD: no kidney disease; NSAID: nonsteroidal anti-inflammatory drug; PCT: procalcitonin; PN: partial nephrectomy; PTA: prothrombin activity; SHAP: Shapley Additive Explanations; TBIL: total bilirubin; WBC: white blood cell.

Sample individualized predictions with their explanations are shown in Figure 3. The AKD and CKD models, respectively, present the top 10 and top 5 features. We selected 4 random samples from the test set and analyzed them using both the SHAP and LIME algorithms. For example, Figure 3D presents an individualized explanation for a case where the actual and predicted outcomes are both CKD. The notably high predicted probability for CKD (*P*=.97) primarily stemmed from several incremental factors, including a low baseline eGFR level (39.36 mL/min/1.73 m^2^), postoperative complications of AKD with AKI, clear cell pathology, and a history of antibiotic use, despite a normal white blood cell level (3.94×10^9^/L). The SHAP force plot revealed minor deviations in the top 5 features for predicting this patient, highlighting the greater significance of γ-glutamyl transferase over albumin-globulin (AG) ratio.

DAG is a type of causal diagram comprising nodes representing features and arrows representing causal relationships between the features. Since the importance of features does not necessarily reflect causality, we designated only AKD and CKD as end points (nodes with only inward-pointing arrows) in the DAGs, without designating source nodes (nodes with only outward-pointing arrows). Given that analyzing all features (over 90) would lead to an excessively complex causal structure, we limited the analysis to the top 20 features for AKD and CKD. During the investigation of AKD as the outcome, we observed direct links from features such as AKI grade, operative duration, systolic blood pressure, Hb, antibiotic, baseline eGFR, urine protein, and hematocrit to AKD, indicating potential direct causality (Figure S8 in Multimedia Appendix 1). All these features, except for antibiotics, are among the top 10 features for AKD prediction. We discovered that the trajectories of renal function, pathology, and baseline eGFR exhibit potential direct causal relationships with CKD, and they also rank among the top 5 features in the CKD model (Figure S9 in Multimedia Appendix 1). Age did not exert a direct effect on CKD but influenced it indirectly through its impact on pathology and baseline eGFR. Despite AG and procedure being within the top 10 features for CKD, our analysis did not reveal a causal link to CKD, suggesting that while there was a correlation between AG or procedure and CKD, they were causally independent.

### Web-Based Calculator

Since the LightGBM model proved to be the most effective in our study, we developed a web-based calculator using the “Streamlit” application to predict both AKD and CKD with this model. Restricting the LightGBM model to only the top 10 and top 5 features did not diminish predictive performance for the AKD and CKD models (AUROC: 0.94 vs 0.97 in AKD prediction and 0.94 vs 0.96 in CKD prediction). For ease of use, we constructed a web-based calculator using the top 10 and top 5 features to predict AKD and CKD, respectively. You can access this calculator at Streamlit [30].

## Discussion

### Principal Findings

Our exploration into the use of ML techniques to predict and elucidate outcomes in patients undergoing nephrectomy was instigated by an amplified emphasis on the long-term renal functional prognosis, the accessibility of intricate data within the electronic health record system, and the maturation of interpretable predictive models. Among patients who underwent nephrectomy, 28.9% (n=451) developed AKI or AKD. Specifically, 7.5% (n=117) of patients developed AKD in conjunction with AKI, 13.7% (n=213) experienced recovery from AKI, and 7.8% (n=121) developed subacute AKD. The incidence rate of CKD was 10.6% (n=165). We formulated a diverse array of ML models with a focus on AKD and CKD prognosis. Among these models, LightGBM exhibited the most robust predictive prowess, achieving an AUROC of 0.97 for AKD prediction and 0.96 for CKD prediction. Our research used SHAP values and the LIME algorithm to interpret the decision-making process from both global and instance-based perspectives. Additionally, we used DAG to further visualize the potential causal relationships between features and outcomes. In consideration of clinical applicability, we further developed a web application that uses the final prediction model to estimate AKD and CKD risks.

### Comparison to Prior Work and Implications

Assessment of renal injury risk following nephrectomy has predominantly concentrated on AKI and CKD, with limited attention to the recovery of renal function within 7-90 days post-AKI and its enduring consequences [5,31-33]. Our prior research has unveiled discernible distinctions in the predicted risk factors between AKI and AKD. Specifically, AKD is associated with a notably elevated risk of de novo CKD development when contrasted with AKI [34]. This study encompassed all patients who were hospitalized, with no specific subgroup analysis conducted for those undergoing nephrectomy. Hu et al [35] initially developed a predictive model for postnephrectomy AKD, using an LR model to assess predicted risk factors associated with renal injury within 3 months following nephrectomy. Nevertheless, this study did not differentiate between AKI recover, subacute AKD, and AKD with AKI. Furthermore, it did not investigate the long-term outcomes for patients experiencing these distinct renal function trajectories. In our study, we found a significant association between trajectories of renal function and the onset and progression of CKD. Specifically, the coexistence of AKD with AKI led to a CKD incidence rate of 35.9% (n=42), nearly 1.5 times higher than that observed in patients with subacute AKD (AKD without AKI). Meanwhile, the CKD incidence rate was 11.3% (n=24) for individuals who had recovered from AKI and 6% (n=67) for those with normal kidney function. Among patients with kidney injury, nearly one-third experienced subacute AKD, which did not meet the criteria for either AKI or CKD diagnosis. These individuals are frequently overlooked in the early stages due to the modest changes in renal function they exhibit; however, their risk of developing CKD is significantly elevated when compared to both patients with NKD and those who have recovered from AKI. As such, the presence of AKD serves as a critical link between AKI and CKD, aiding in the assessment of declining renal function and prognosis.

Currently, LR is the most widely used model for predicting kidney injury risk in patients undergoing nephrectomy, with limited application of ML algorithms [36,37]. Lee et al [38] used various ML algorithms to formulate a risk prediction model for AKI after nephrectomy, identifying that the LightGBM model outperforms others in terms of predictive accuracy. Compared to LR, LightGBM demonstrates enhanced speed, more efficient memory use, and superior parallel processing capabilities, which allow it to more effectively manage nonlinear relationships, large datasets, and high-dimensional data [39]. Our study has undertaken a thorough evaluation of the predictive abilities of several ML models, with LightGBM emerging as the most effective in forecasting high-risk AKD and CKD cases, alongside precisely pinpointing individual predicted risk factors. Early alerts assist in promptly notifying clinicians to undertake vigilant monitoring of patients at high risk. Addressing manageable predicted risk factors early, such as Hb, systolic blood pressure, and total bilirubin, presents a considerable opportunity to lower the occurrence of AKD and CKD, thereby enhancing patient outcomes.

Given our emphasis on interpretability, our methodology entails a thorough interpretation of the entire predictive algorithm, exploring the potential causal relationships between major features. First, we generate global-level diagrams that elucidate the contributions of each feature to the model’s output along with interactions among key features. Features denoting acute injury, such as surgical factors and AKI grade, exert a significant influence on AKD. Baseline eGFR and trajectories of renal function constitute pivotal features affecting CKD. Features such as advanced age or clear cell carcinoma may be associated with an elevated CKD risk. While these attributes are generally nonmodifiable, augmenting the frequency of follow-up visits for individuals with these characteristics can effectively facilitate the early detection of renal function deterioration. Second, this study delineates the decision-making process for each patient. The examples depicted in Figure 3 elucidate the predominant feature compositions among patients exhibiting diverse predicted probabilities of AKD or CKD. Using SHAP force plots and LIME plots amplifies the individualization and transparency of the decision-making process, thereby alleviating the black-box issue inherent in the model’s prediction process. Finally, DAGs were used to delve deeper into the potential causal relationships between features and outcomes. It was found that most of the top 10 features identified by SHAP values have the potential to directly or indirectly influence the occurrence of AKD or CKD.

For the sake of enhancing user convenience, we have developed web-based prediction tools for both AKD and CKD. Users can effortlessly input the values of their chosen features to calculate the probabilities of AKD and CKD following nephrectomy. Our research marks a pioneering effort in constructing web-based prediction tools for postnephrectomy AKD and CKD, which can assist clinicians in identifying high-risk individuals and risk factors. Given the clinical feasibility and straightforward accessibility of features derived from routine medical records, our models are eminently suitable for seamless integration into daily clinical practice.

### Limitations and Future Directions

The study exhibits several limitations. First, the web-based prediction tool is crafted to assist clinicians in discerning individuals with elevated risk of AKD and CKD rather than serving as a replacement for clinical diagnosis. Due to the retrospective nature of data collection, it is crucial to undertake additional validation using an independent population to ensure robust predictive validity across diverse usage scenarios. Second, the collection of urine output data is subjective, and a significant number of values are missing. Consequently, this study refrained from using urine output as a diagnostic criterion for AKI. Third, our study lacks time-variant monitored values among its features. Moving forward, we intend to collect longitudinally monitored data from patients undergoing nephrectomy to enable dynamic prediction of AKD and CKD before their clinical identification. Finally, DAGs visually represent the potential causal relationships between features and outcomes. This underscores the need to further explore and quantify the causal mechanisms in future work.

### Conclusions

This study has developed prediction models that accurately estimate the risk of AKD and CKD following nephrectomy. These models provide interpretability from both global and instance-based perspectives. We recommend the use of the AKD criterion in clinical practice due to its superior accuracy in predicting prognosis, particularly the development of CKD.

## Appendix

Multimedia Appendix 1 Quantile-quantile plots, model performance, Shapley Additive Explanations plots, and directed acyclic graph of the study.

Multimedia Appendix 2 Descriptive statistics for the cohorts; performance of models.

## Abbreviations

AG: albumin-globulin
AKD: acute kidney disease
AKI: acute kidney injury
AUROC: area under the receiver operating characteristic curve
CKD: chronic kidney disease
DAG: directed acyclic graph
eGFR: estimated glomerular filtration rate
Hb: hemoglobin
LightGBM: Light Gradient-Boosting Machine
LIME: Local Interpretable Model-Agnostic Explanations
LR: logistic regression
ML: machine learning
NKD: no kidney disease
PN: partial nephrectomy
RF: random forest
RN: radical nephrectomy
Scr: serum creatinine
SHAP: Shapley Additive Explanations

## Data Availability

The datasets analyzed during this study are available from the corresponding author upon reasonable request.
