# Supplementary material for: Personalized Prediction of Long-Term Renal Function Prognosis Following Nephrectomy Using Interpretable Machine Learning Algorithms: Case-Control Study
Source: JMIR Med Inform. 2024 Sep 20;12:e52837. doi: 10.2196/52837 (PMC11452755; doi:10.2196/52837)
Supplement: Multimedia Appendix 1 [file medinform_v12i1e52837_app1.docx]

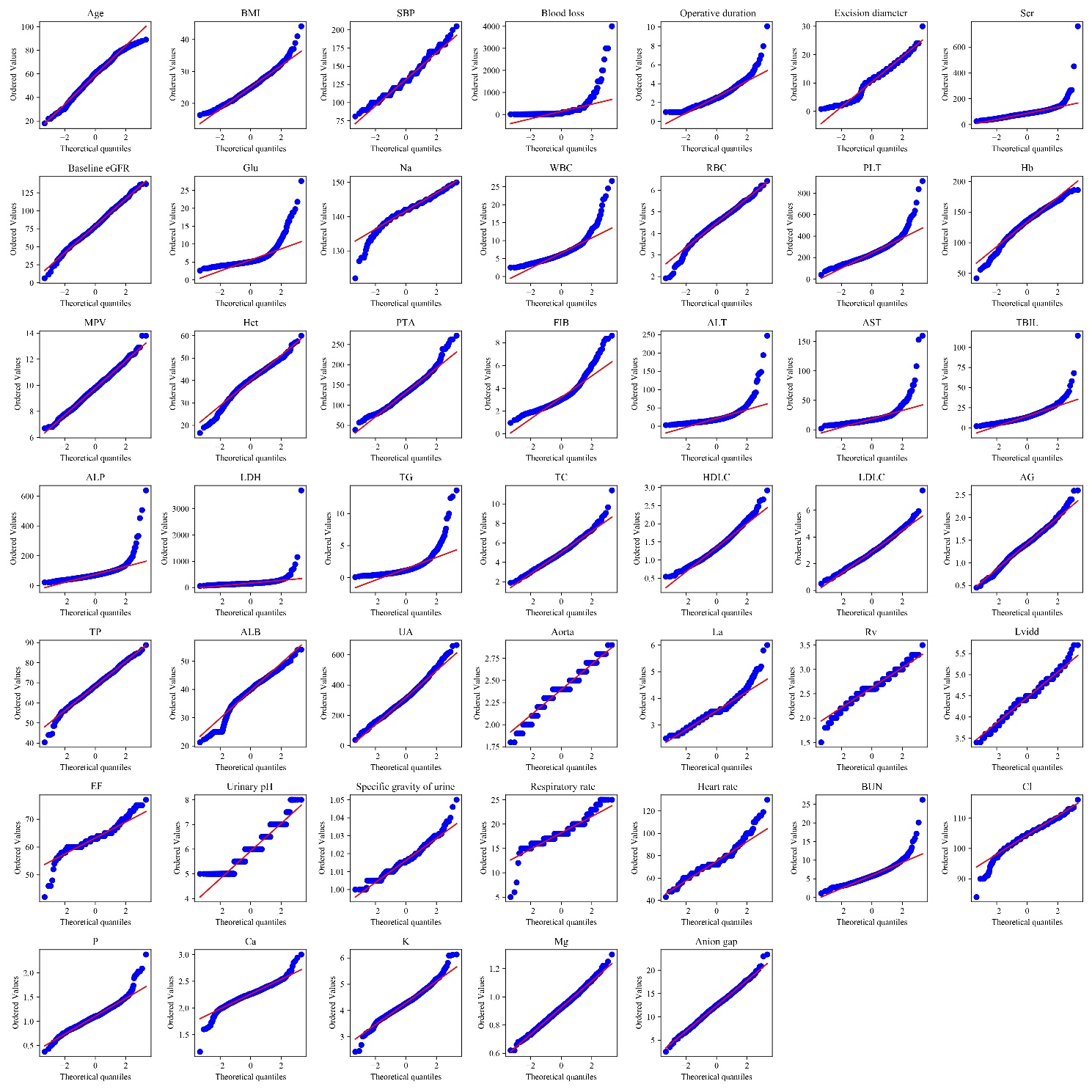


**Figure S1.** Quantile-Quantile plots for assessing the normality of continuous features.


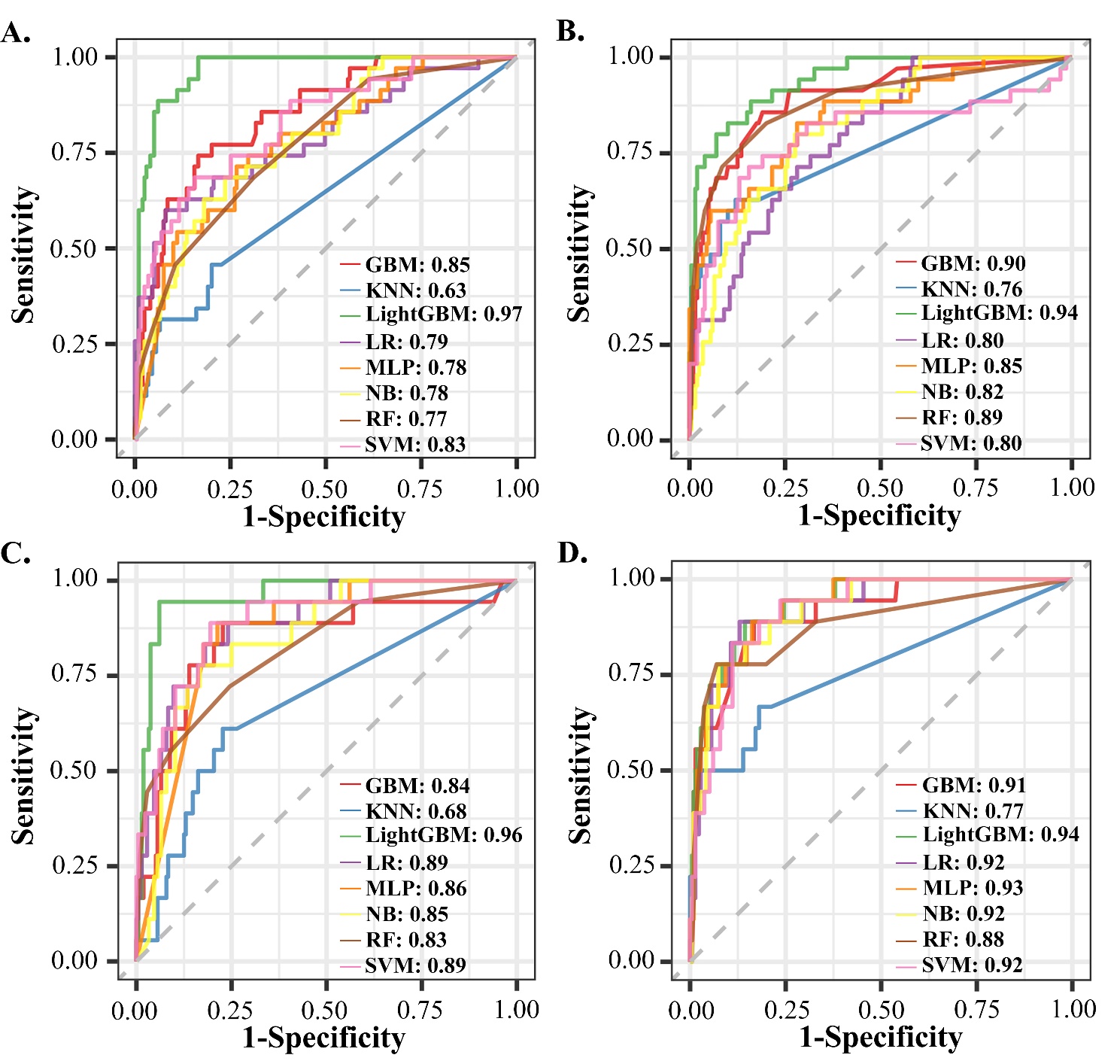


**Figure S2.** ROC curves and AUROC values for 8 machine learning models predicting AKD (A-B) and CKD (C-D) using the test set. A. Prediction of AKD using all features. B. Prediction of AKD using the top 10 features. C. Prediction of CKD using all features. D. Prediction of CKD using the top 5 features.


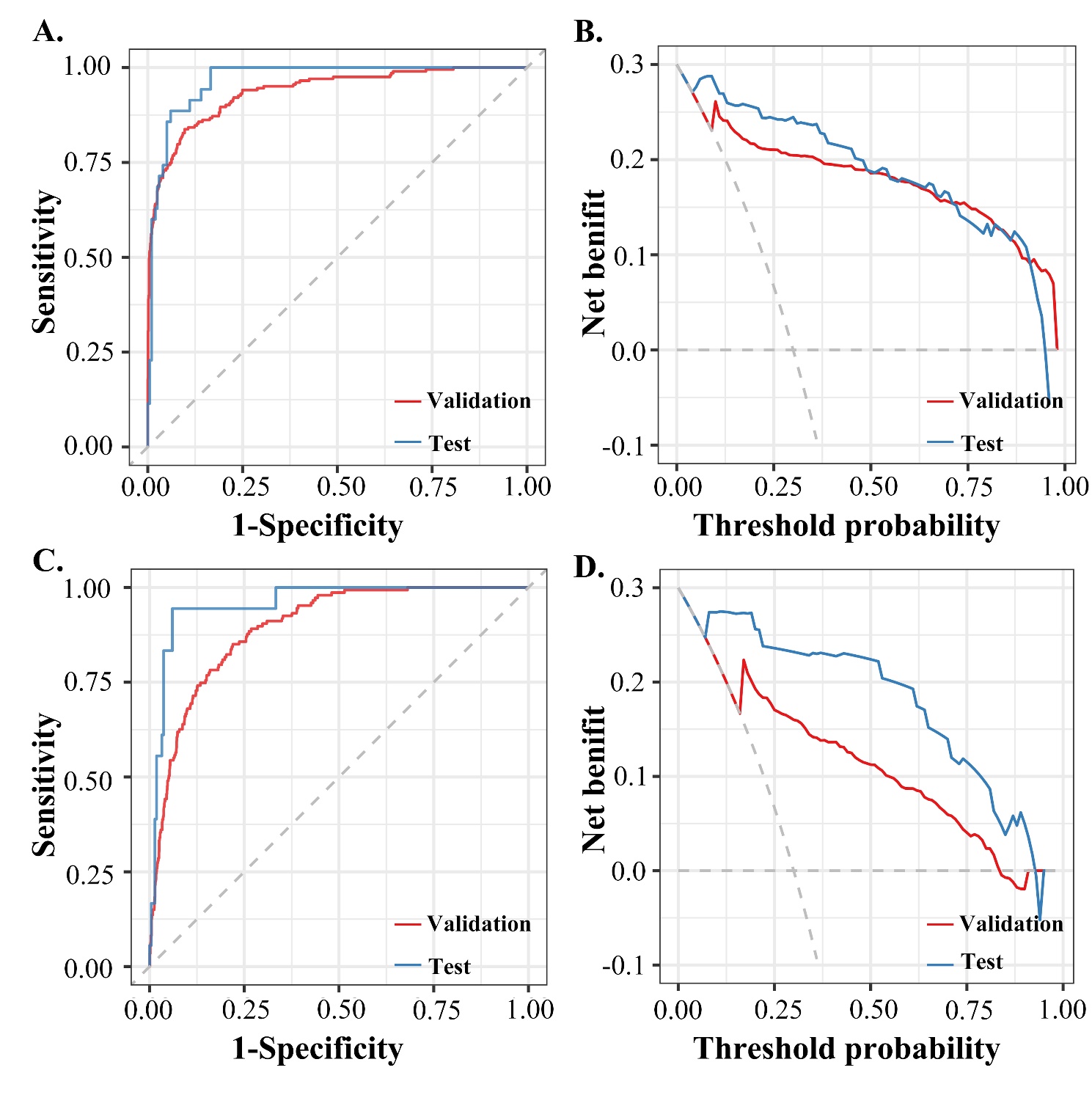


**Figure S3.** ROC and DCA curves illustrating the performance of lightGBM models in predicting AKD (A-B) and CKD (C-D) using all features.


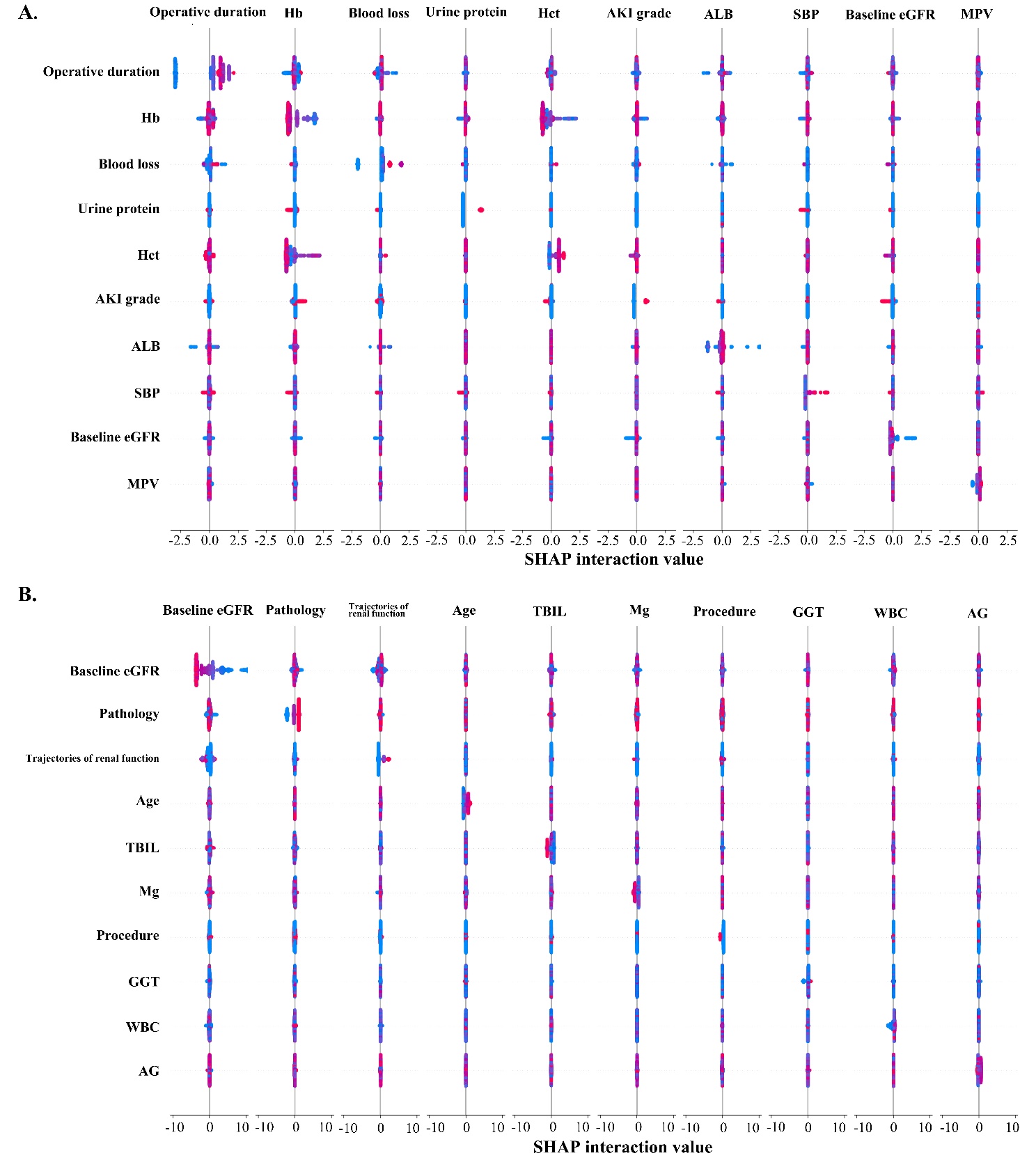


**Figure S4.** SHAP interaction plots depicting the interactions among the top 10 features of the lightGBM models for AKD (A) and CKD (B).


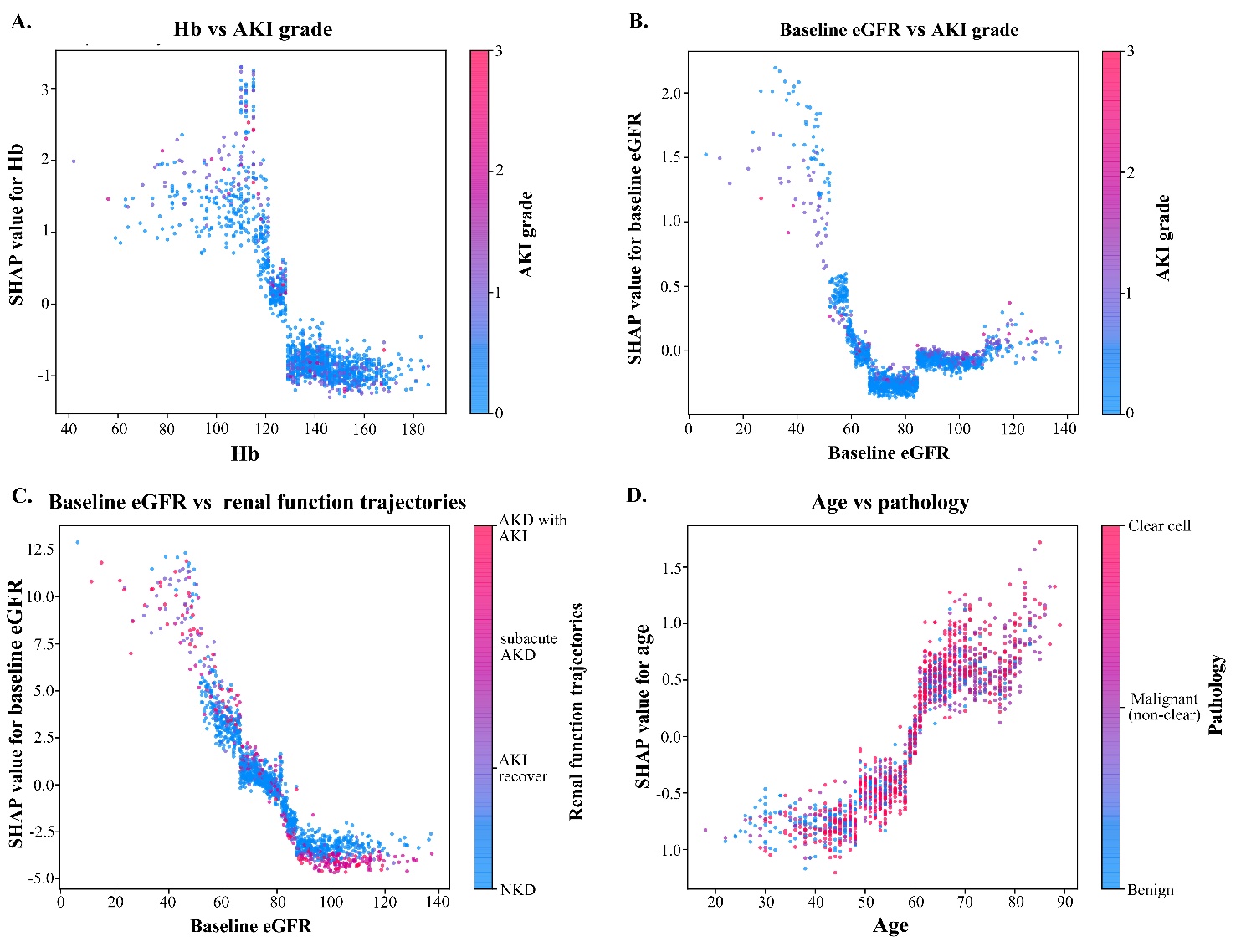


**Figure S5.** The SHAP dependence plots illustrate the correlations between key features in the prediction of AKD (A-B) and CKD (C-D). A. depicts the correlation between Hb and AKI grade in predicting AKD. The x-axis represents the actual values of Hb, whereas the y-axis shows the SHAP values for Hb, with values above zero suggesting an increased risk of AKD. Each dot symbolizes a unique case, with the color transitioning from blue to red to indicate increasing AKI grades. Specifically, the influence of AKI grade on the probability of AKD varies across Hb levels. Among patients with lower Hb levels, higher AKI grades are associated with a significant increase in the risk of AKD. Conversely, this correlation is less pronounced in patients with higher Hb levels. B. depicts the correlation between baseline eGFR and AKI grade in predicting AKD. Among patients with lower baseline eGFR levels, higher AKI grades are associated with a significant increase in the risk of AKD. C. depicts the correlation between baseline eGFR and renal function trajectories in predicting CKD. For patients presenting with a baseline eGFR below 80, postoperative complications, such as AKD with AKI, subacute AKD, or AKI recover, markedly elevate the risk of developing CKD. D. depicts the correlation between Age and pathology in predicting CKD.


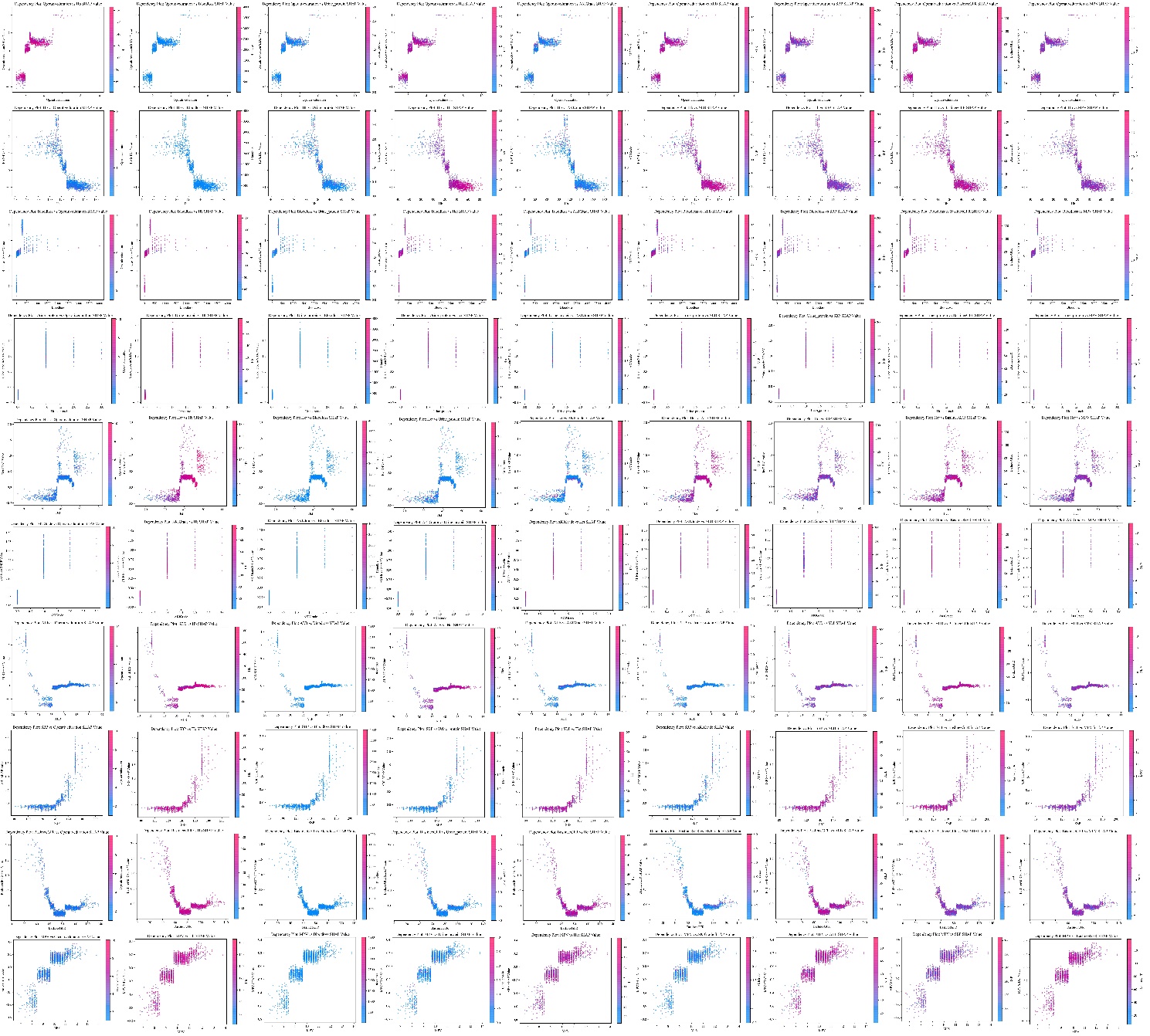


**Figure S6.** The SHAP dependence plots elucidating correlations through pairwise combinations of the top 10 features in predicting AKD.


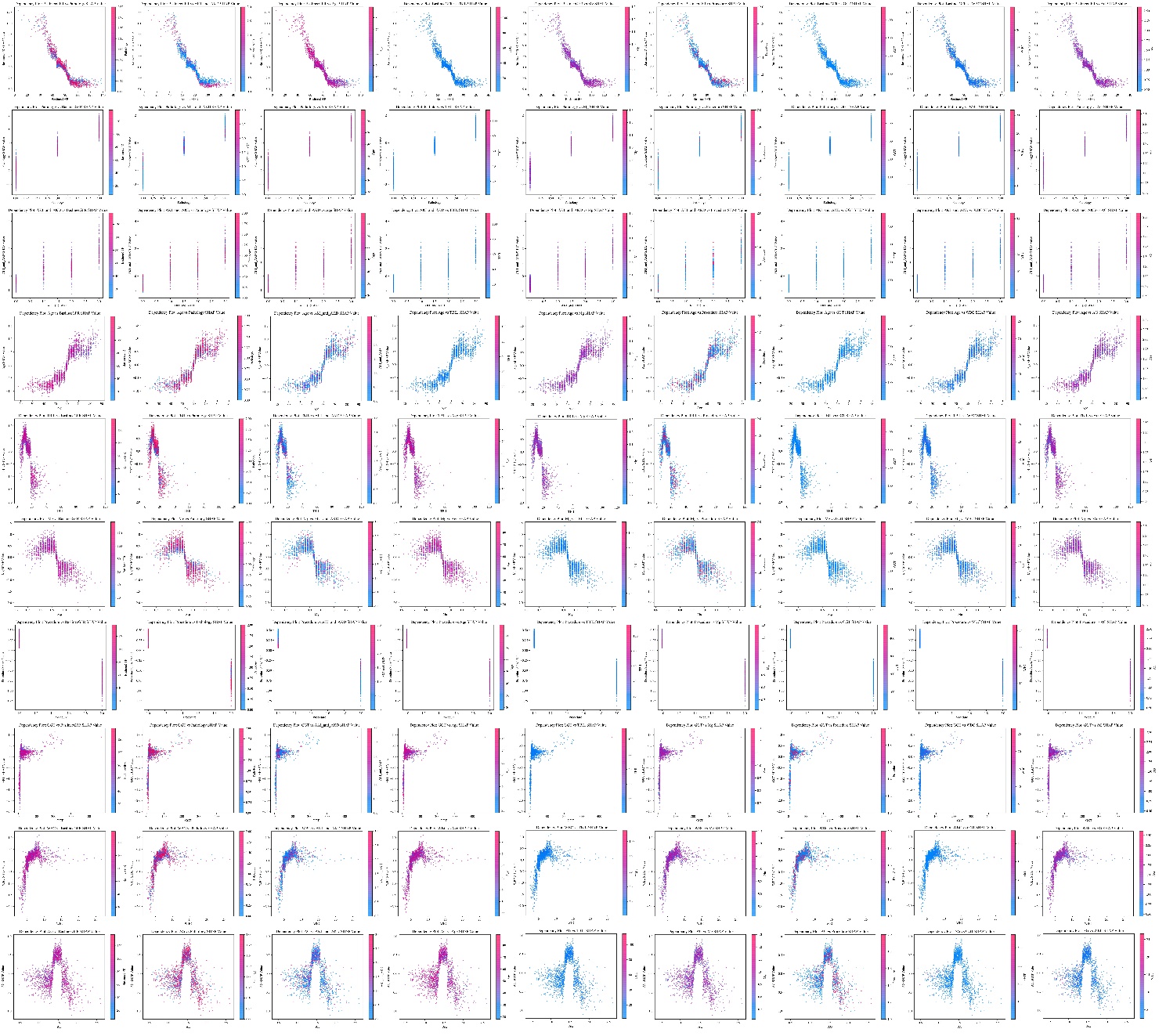


**Figure S7.** The SHAP dependence plots elucidating correlations through pairwise combinations of the top 10 features in predicting CKD.


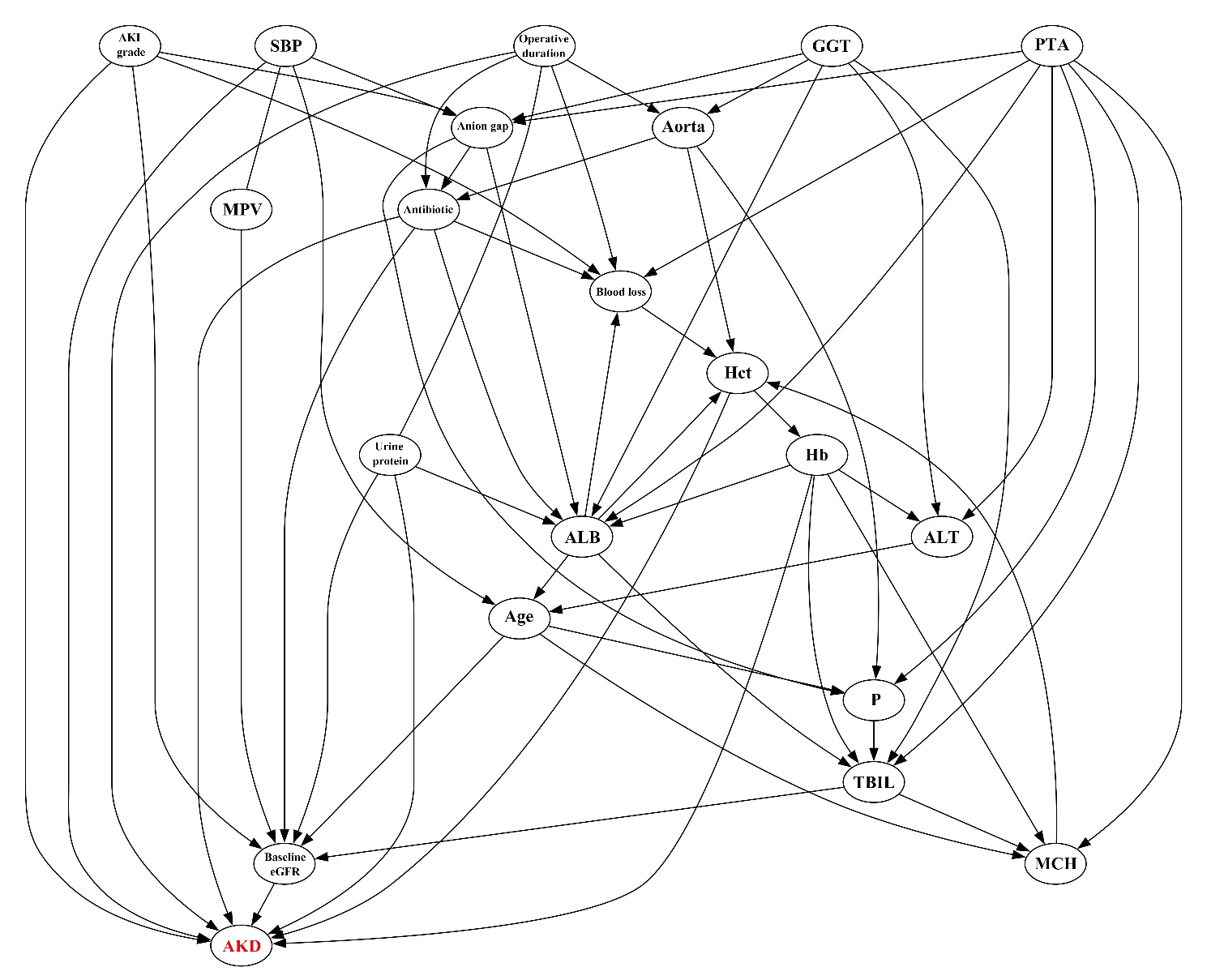


**Figure S8.** DAG representing the causal assumptions used top 20 features in AKD prediction. Each feature represents as a node, and features that are related are connected by directed edges (arrows): a direct effect of one feature over the other is represented by an arrowhead. The absence of an arrow between 2 features represents the assumption that there is no direct causal effect.


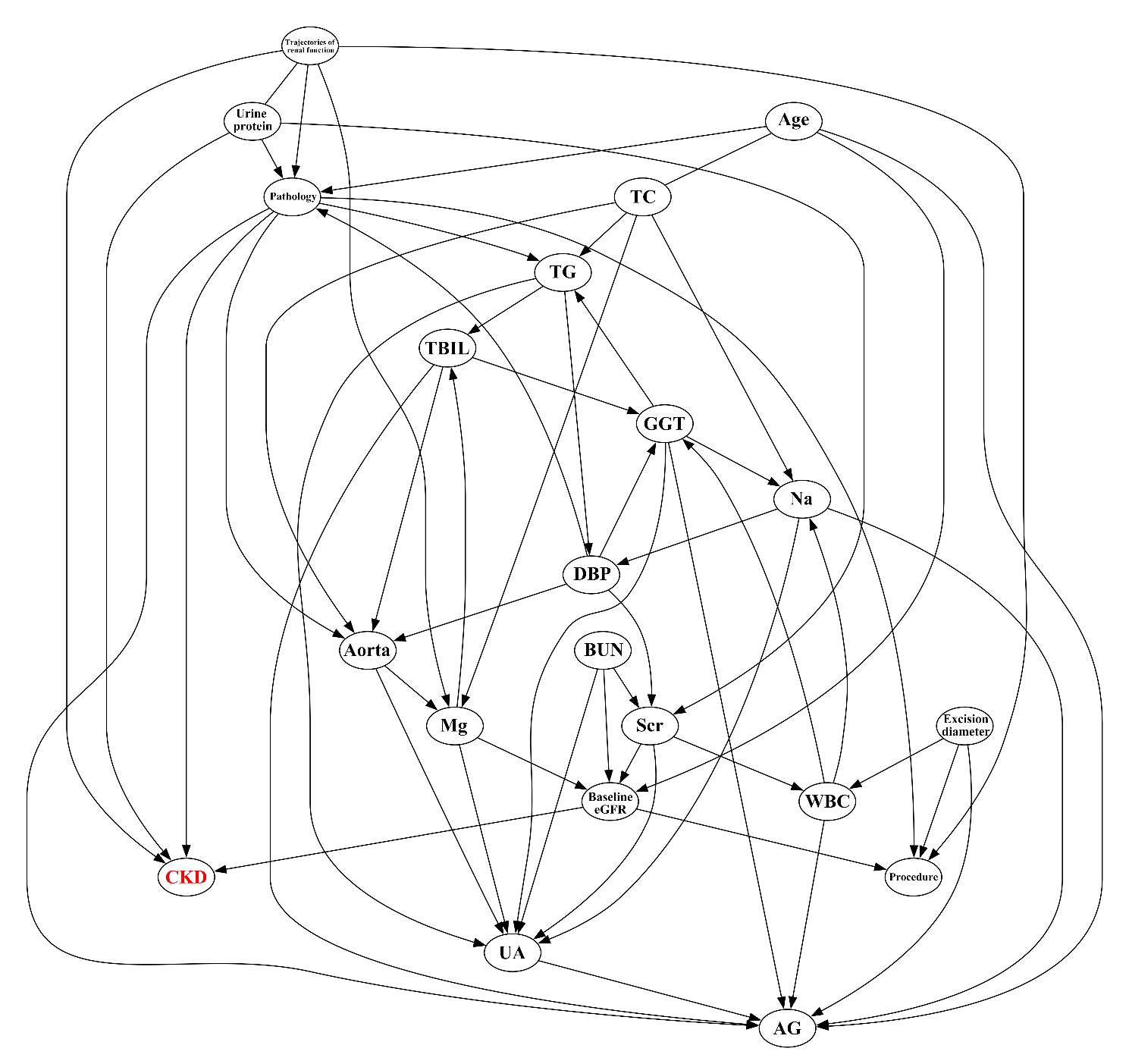


**Figure S9.** DAG representing the causal assumptions used top 20 features in CKD prediction.
