# Supplementary material for: Personalized Prediction of Long-Term Renal Function Prognosis Following Nephrectomy Using Interpretable Machine Learning Algorithms: Case-Control Study
Source: JMIR Med Inform. 2024 Sep 20;12:e52837. doi: 10.2196/52837 (PMC11452755; doi:10.2196/52837)
Supplement: Multimedia Appendix 2 [file medinform_v12i1e52837_app2.docx]

**Table S1.** Baseline characteristics of patients with NKD and acute/subacute renal impairment (mean±SD; [Q1, Q3]; n (%))*.

| Features | | NKD (N=1108) | Acute/ subacute renal impairment | | | | |
| --- | --- | --- | --- | --- | --- | --- | --- |
|  |  |  | subacute AKD (N=121) | AKI recover (N=213) | AKD with AKI (N=117) | Total (N=451) | P-value |
| Age (years) | | 58.14 ± 12.14 | 63.25 ± 12.05 | 58.08 ± 13.22 | 66.01 ± 11.31 | 61.53 ± 12.87 | < .001 |
| Male (%) | | 622 (56.14) | 72 (59.50) | 156 (73.24) | 64 (54.70) | 292 (64.75) | < .001 |
| BMI (kg/m2) | | 24.87 ± 3.37 | 25.03 ± 3.76 | 25.31 ± 3.45 | 25.68 ± 3.49 | 25.33 ± 3.54 | .02 |
| SBP (mmHg) | | 130.04 ± 16.77 | 138.96 ± 24.52 | 130.28 ± 17.54 | 144.58 ± 21.86 | 136.32 ± 21.57 | < .001 |
| Blood transfusion (%) | | 77 (6.95) | 13 (10.74) | 12 (5.63) | 15 (12.82) | 40 (8.87) | .23 |
| Smokers (%) | | 385 (34.75) | 59 (48.76) | 77 (36.15) | 49 (41.88) | 185 (41.02) | .02 |
| Drinkers (%) | | 320 (28.88) | 31 (25.62) | 79 (37.09) | 34 (29.06) | 144 (31.93) | .26 |
| Medical insurance (%) | | 978 (88.27) | 109 (90.08) | 183 (85.92) | 106 (90.60) | 398 (88.25) | 1 |
| Fever (%) | | 40 (3.61) | 7 (5.79) | 6 (2.82) | 3 (2.56) | 16 (3.55) | 1 |
| Respiratory rate | | 18 [17, 19] | 18 [17, 19] | 18 [17, 19] | 18 [17, 20] | 18 [17, 19] | .11 |
| Heart rate | | 75.15 ± 8.93 | 76.62 ± 11.09 | 74.21 ± 8.50 | 74.16 ± 9.57 | 74.84 ± 9.57 | .55 |
| **Procedure, n (%)** | | | | | | | |
|  | RN | 752 (67.87) | 103 (85.12) | 174 (81.69) | 102 (87.18) | 379 (84.04) | < .001 |
|  | PN | 356 (32.13) | 18 (14.88) | 39 (18.31) | 15 (12.82) | 72 (15.96) |  |
|  | Approach, n (%) |  |  |  |  |  | .13 |
|  | laparotomy | 833 (75.18) | 86 (71.07) | 159 (74.65) | 74 (63.25) | 319 (70.73) |  |
|  | laparoscopy | 215 (19.40) | 27 (22.31) | 39 (18.31) | 35 (29.91) | 101 (22.39) |  |
|  | Da Vinci Surgery | 60 (5.42) | 8 (6.61) | 15 (7.04) | 8 (6.84) | 31 (6.87) |  |
|  | Pathology, n (%) |  |  |  |  |  | < .001 |
|  | Benign | 298 (26.90) | 22 (18.18) | 21 (9.86) | 16 (13.68) | 59 (13.08) |  |
|  | Malignant (non-clear) | 282 (25.45) | 43 (35.54) | 61 (28.64) | 45 (38.46) | 149 (33.04) |  |
|  | Clear cell | 528 (47.65) | 56 (46.28) | 131 (61.50) | 56 (47.86) | 243 (53.88) |  |
|  | Blood loss (ML) | 50 [20, 150] | 100 [50, 200] | 50 [20, 150] | 150 [100, 300] | 100 [25, 200] | < .001 |
|  | Excision diameter (cm) | 11 [5, 13] | 12 [9, 14] | 12 [10, 14] | 12 [10, 15] | 12 [10, 14] | < .001 |
|  | Operative duration (hours) | 2.33 [1.92, 2.92] | 2.67 [2.50, 3.50] | 2.50 [1.92, 3.07] | 2.83 [2.50, 3.50] | 2.58 [2.25, 3.22] | < .001 |
| **Laboratory tests** | | | | | | | |
|  | WBC (×109/L) | 6.0 [5.0, 7.2] | 6.8 [5.8, 8.5] | 6.4 [5.2, 7.5] | 6.2 [4.8, 7.6] | 6.4 [5.2, 7.8] | < .001 |
|  | RBC (×1012/L) | 4.51 ± 0.55 | 4.37 ± 0.59 | 4.61 ± 0.55 | 4.13 ± 0.67 | 4.42 ± 0.63 | .01 |
|  | PLT (×109/L) | 232 [193, 278] | 246 [202, 283] | 228 [194, 273] | 223 [182, 266] | 233 [193, 276] | .92 |
|  | Hb (g/L) | 135.43 ± 19.34 | 123.43 ± 20.40 | 139.27 ± 19.51 | 119.94 ± 21.91 | 130.00 ± 22.20 | < .001 |
|  | MPV (fL) | 9.77 ± 1.05 | 9.87 ± 1.00 | 9.81 ± 0.98 | 9.87 ± 1.05 | 9.84 ± 1.00 | .19 |
|  | Hct (%) | 40.25 ± 5.48 | 38.61 ± 5.80 | 41.57 ± 5.25 | 36.53 ± 6.40 | 39.47 ± 6.09 | .01 |
|  | PTA (%) | 132.31 ± 31.53 | 123.10 ± 30.23 | 132.96 ± 24.16 | 128.85 ± 28.75 | 129.25 ± 27.36 | .07 |
|  | FIB (g/L) | 2.96 [2.5, 3.51] | 3.22 [2.77, 4.01] | 3.00 [2.60, 3.45] | 3.22 [2.76, 3.71] | 3.13 [2.67, 3.67] | < .001 |
|  | Scr (μmol/L) | 86 [75, 97] | 90 [77, 110] | 80 [64, 94] | 85 [66, 110] | 83 [67, 101] | .10 |
|  | BUN (mmol/L) | 5.7 [4.7, 6.7] | 5.9 [4.7, 7.8] | 5.6 [4.7, 6.7] | 5.9 [4.8, 7.5] | 5.8 [4.7, 7.1] | .11 |
|  | UA (μmol/L) | 311.27 ± 87.63 | 334.16 ± 99.72 | 324.43 ± 87.40 | 332.70 ± 91.64 | 329.19 ± 91.85 | < .001 |
|  | Basline eGFR (ml/min/1.73 m2) | 79.30 ± 16.01 | 69.32 ± 23.20 | 88.17 ± 20.54 | 73.12 ± 25.52 | 79.21 ± 24.17 | .93 |
|  | ALT (U/L) | 18 [13, 24] | 17 [12, 23] | 18 [13, 24] | 16 [13, 23] | 17 [13, 24] | .26 |
|  | AST (U/L) | 17 [14, 20] | 16 [14, 20] | 17 [14, 20] | 17 [13, 20] | 17 [14, 20] | .71 |
|  | TBIL (μmol/L) | 13.1 [10.1, 17.62] | 13.1 [9.9, 17.5] | 14.0 [10.1, 18.7] | 12.9 [9.3, 16.6] | 13.2 [10.0, 17.9] | .02 |
|  | ALP (U/L) | 68 [57, 83] | 75 [60, 89] | 68 [57, 84] | 71 [60, 89] | 71 [58, 87] | < .001 |
|  | LDH (U/L) | 152 [137, 176] | 153 [132, 178] | 160 [140, 182] | 161 [141, 193] | 159 [138, 183] | .01 |
|  | TG (mmol/L) | 1.1 [0.8, 1.6] | 1.1 [0.9, 1.6] | 1.1 [0.8, 1.7] | 1.2 [0.9, 1.7] | 1.1 [0.8, 1.7] | .18 |
|  | TC (mmol/L) | 5.06 ± 1.07 | 4.93 ± 1.29 | 4.94 ± 1.05 | 4.93 ± 1.19 | 4.93 ± 1.15 | .05 |
|  | HDLC (mmol/L) | 1.36 ± 0.34 | 1.28 ± 0.32 | 1.32 ± 0.31 | 1.28 ± 0.35 | 1.30 ± 0.32 | < .001 |
|  | LDLC (mmol/L) | 2.90 ± 0.80 | 2.87 ± 0.92 | 2.89 ± 0.81 | 2.78 ± 0.75 | 2.85 ± 0.82 | .34 |
|  | TP (g/L) | 68.59 ± 6.04 | 68.63 ± 6.43 | 68.10 ± 5.95 | 66.87 ± 6.65 | 67.92 ± 6.29 | .05 |
|  | ALB (g/L) | 39.97 ± 4.43 | 37.87 ± 7.15 | 39.96 ± 4.16 | 36.21 ± 6.70 | 38.42 ± 5.98 | < .001 |
|  | Glu (mmol/L) | 5.0 [4.6, 5.8] | 5.2 [4.7, 6.2] | 5.1 [4.6, 5.7] | 5.4 [4.7, 6.4] | 5.1 [4.6, 5.9] | .09 |
|  | Anion gap (mmol/L) | 12.28 ± 2.78 | 12.56 ± 2.55 | 12.78 ± 2.58 | 12.51 ± 2.57 | 12.65 ± 2.57 | .01 |
|  | Cl (mmol/L) | 104.41 ± 3.09 | 104.04 ± 4.08 | 104.22 ± 2.82 | 104.55 ± 3.86 | 104.26 ± 3.47 | .40 |
|  | P (mmol/L) | 1.10 ± 0.18 | 1.09 ± 0.19 | 1.11 ± 0.20 | 1.12 ± 0.21 | 1.11 ± 0.20 | .72 |
|  | Ca (mmol/L) | 2.3 [2.2, 2.3] | 2.3 [2.2, 2.4] | 2.3 [2.2, 2.4] | 2.3 [2.1, 2.4] | 2.3 [2.2, 2.4] | .20 |
|  | K (mmol/L) | 4.28 ± 0.39 | 4.25 ± 0.45 | 4.36 ± 0.44 | 4.26 ± 0.54 | 4.30 ± 0.47 | .26 |
|  | Na (mmol/L) | 141.69 ± 2.64 | 141.30 ± 3.02 | 141.74 ± 2.47 | 141.24 ± 3.36 | 141.49 ± 2.88 | .18 |
|  | Mg (mmol/L) | 0.92 ± 0.09 | 0.93 ± 0.10 | 0.95 ± 0.09 | 0.92 ± 0.09 | 0.94 ± 0.09 | < .001 |
| **Urinalysis** | | | | | | | |
|  | pH | 5.94 ± 0.58 | 5.99 ± 0.64 | 5.80 ± 0.59 | 5.85 ± 0.58 | 5.86 ± 0.61 | .02 |
|  | Specific gravity | 1.02 ± 0.01 | 1.02 ± 0.01 | 1.02 ± 0.01 | 1.01 ± 0.01 | 1.02 ± 0.01 | .83 |
|  | Protein | 149 (13.45) | 62 (51.24) | 29 (13.62) | 67 (57.26) | 158 (35.03) | < .001 |
|  | Nitrites | 38 (3.43) | 5 (4.13) | 5 (2.35) | 5 (4.27) | 15 (3.33) | 1 |
|  | Urobilinogen | 29 (2.62) | 6 (4.96) | 9 (4.23) | 6 (5.13) | 21 (4.66) | .06 |
|  | Glucose | 144 (13.00) | 20 (16.53) | 13 (6.10) | 29 (24.79) | 62 (13.75) | .75 |
|  | Hematuria | 699 (63.09) | 95 (78.51) | 119 (55.87) | 89 (76.07) | 303 (67.18) | .14 |
| **Echocardiography** | | | | | | | |
|  | EF | 63 [62, 65] | 63 [61, 64] | 62 [60, 64] | 63 [60, 64] | 63 [60, 64] | < .001 |
|  | Aorta | 2.39 ± 0.15 | 2.41 ± 0.15 | 2.41 ± 0.15 | 2.41 ± 0.16 | 2.41 ± 0.15 | .02 |
|  | La | 3.52 ± 0.35 | 3.60 ± 0.42 | 3.55 ± 0.34 | 3.63 ± 0.50 | 3.58 ± 0.41 | < .001 |
|  | Rv | 2.62 ± 0.21 | 2.65 ± 0.20 | 2.63 ± 0.22 | 2.66 ± 0.19 | 2.64 ± 0.21 | .05 |
|  | Lvidd | 4.46 ± 0.30 | 4.48 ± 0.31 | 4.44 ± 0.30 | 4.50 ± 0.34 | 4.47 ± 0.31 | .68 |
|  | E/A > 1, n (%) | 115 (10.38) | 12 (9.92) | 29 (13.62) | 5 (4.27) | 46 (10.20) | .99 |
| **Comorbidities, n (%)** | | | | | | | |
|  | Renal calculi | 158 (14.26) | 18 (14.88) | 4 (1.88) | 9 (7.69) | 31 (6.87) | < .001 |
|  | Arrhythmia | 13 (1.17) | 2 (1.65) | 0 (0.00) | 1 (0.85) | 3 (0.67) | .53 |
|  | Peptic ulcer | 47 (4.24) | 2 (1.65) | 10 (4.69) | 3 (2.56) | 15 (3.33) | .49 |
|  | Fatty liver | 52 (4.69) | 3 (2.48) | 10 (4.69) | 5 (4.27) | 18 (3.99) | .64 |
|  | Hydronephrosis | 161 (14.53) | 15 (12.40) | 7 (3.29) | 12 (10.26) | 34 (7.54) | < .001 |
|  | Diabetes mellitus | 122 (11.01) | 28 (23.14) | 23 (10.80) | 29 (24.79) | 80 (17.74) | < .001 |
|  | CHD | 77 (6.95) | 13 (10.74) | 14 (6.57) | 20 (17.09) | 47 (10.42) | .03 |
|  | Hypertension | 311 (28.07) | 55 (45.45) | 61 (28.64) | 61 (52.14) | 177 (39.25) | < .001 |
|  | Anemia | 241 (21.75) | 66 (54.55) | 34 (15.96) | 71 (60.68) | 171 (37.92) | < .001 |
|  | Dyslipidemia | 429 (38.72) | 53 (43.80) | 83 (38.97) | 59 (50.43) | 195 (43.24) | .11 |
|  | hyperuricemia | 162 (14.62) | 30 (24.79) | 36 (16.90) | 27 (23.08) | 93 (20.62) | < .001 |
|  | Obesity | 191 (17.24) | 33 (27.27) | 44 (20.66) | 34 (29.06) | 111 (24.61) | < .001 |
| **Medications, n (%)** | | | | | | | |
|  | β-blocker | 433 (39.08) | 63 (52.07) | 75 (35.21) | 59 (50.43) | 197 (43.68) | .10 |
|  | ACEI/ARB | 97 (8.75) | 25 (20.66) | 19 (8.92) | 22 (18.80) | 66 (14.63) | < .001 |
|  | CCB | 248 (22.38) | 32 (26.45) | 48 (22.54) | 50 (42.74) | 130 (28.82) | .01 |
|  | Aspirin | 163 (14.71) | 28 (23.14) | 36 (16.90) | 36 (30.77) | 100 (22.17) | < .001 |
|  | Statins | 45 (4.06) | 10 (8.26) | 9 (4.23) | 12 (10.26) | 31 (6.87) | .03 |
|  | Antibiotics | 730 (65.88) | 91 (75.21) | 121 (56.81) | 100 (85.47) | 312 (69.18) | .23 |
|  | NSAIDs | 280 (25.27) | 44 (36.36) | 50 (23.47) | 42 (35.90) | 136 (30.16) | .06 |
|  | Diuretics | 260 (23.47) | 40 (33.06) | 70 (32.86) | 65 (55.56) | 175 (38.80) | < .001 |
|  | Antidiabetic medication | 52 (4.69) | 10 (8.26) | 6 (2.82) | 10 (8.55) | 26 (5.76) | .45 |
| **Outcome** | | | | | | | |
|  | AKI, n (%) | 0 (0.00) | 0 (0.00) | 213 (100.00) | 117 (100.00) | 330 (73.17) | < .001 |
|  | AKD, n (%) | 0 (0.00) | 121 (100.00) | 0 (0.00) | 117 (100.00) | 238 (52.77) | < .001 |
|  | CKD, n (%) | 67 (6.05) | 32 (26.45) | 24 (11.27) | 42 (35.90) | 98 (21.73) | < .001 |
|  | LOS, median [Q1, Q3] | 11 [9, 13] | 13 [11, 16] | 10 [8, 11] | 13 [9, 16] | 11 [9, 1r4] | .04 |

* ACEI/ARB: angiotensin-converting enzyme inhibitor/angiotensin receptor blocker; ADA: adenosine deaminase; AKD: acute kidney disease; AKI: acute kidney injury; ALB: albumin; ALP: alkaline phosphatase; ALT: alanine transaminase; AST: aspartate transaminase; BMI: body mass index; BUN: blood urea nitrogen; CCB: calcium channel blocker; CHD: coronary heart disease; CKD: chronic kidney disease; DBP: diastolic blood pressure; EF: ejection fraction; eGFR: estimated glomerular filtration rate; FIB: fibrinogen; GGT: gamma-glutamyl transferase; Glu: blood glucose; Hb: hemoglobin; Hct: hematocrit; HDLC: high-density lipoprotein cholesterol; La: left atrium; LDH: lactate dehydrogenase; LDLC: low-density lipoprotein cholesterol; LOS: length of stay; LPa: lipoprotein a; Lvidd: left ventricular internal dimension in diastole; Lvpwd: left ventricular posterior wall thickness; Lvs: left ventricular septum; MCH: mean corpuscular hemoglobin; MCHC: mean corpuscular hemoglobin concentration; MCV: mean corpuscular volume; MPV: mean Platelet Volume; NSAIDs: non-steroidal anti-inflammatory drugs; PCT: procalcitonin; PLT: platelet; PN: partial nephrectomy; PTA: prothrombin activity; RBC: red blood cell; RN: radical nephrectomy; Rv: right ventricle; SBP: systolic blood pressure; Scr: serum creatinine; SD: standard deviation; TBIL: total bilirubin; TC: total cholesterol; TG: triglyceride; TP: total protein; UA: uric acid; WBC: white blood cell.

**Table S2.** Performance of eight ML models in predicting AKD by leveraging the all features*.

| Target | AUROC | Precision | Recall | Accuracy | F1 score | MCC | BSL |
| --- | --- | --- | --- | --- | --- | --- | --- |
| Validation set |  |  |  |  |  |  |  |
| LightGBM | 0.94 (0.91-0.96) | 0.80 (0.76-0.83) | 0.72 (0.67-0.77) | 0.93 (0.92-0.94) | 0.75 (0.72-0.78) | 0.71 (0.68-0.75) | 0.06 (0.05-0.06) |
| GBM | 0.84 (0.81-0.88) | 0.20 (0.00-0.50) | 0.01 (0.00-0.02) | 0.85 (0.85-0.85) | 0.02 (0.00-0.05) | 0.04 (0.00-0.10) | 0.11 (0.11-0.11) |
| RF | 0.79 (0.75-0.84) | 0.61 (0.47-0.75) | 0.25 (0.16-0.34) | 0.86 (0.85-0.88) | 0.35 (0.23-0.47) | 0.33 (0.21-0.45) | 0.10 (0.09-0.11) |
| KNN | 0.60 (0.57-0.63) | 0.45 (0.34-0.56) | 0.13 (0.08-0.19) | 0.84 (0.83-0.85) | 0.20 (0.13-0.27) | 0.18 (0.11-0.25) | 0.14 (0.13-0.15) |
| MLP | 0.79 (0.73-0.86) | 0.22 (0.01-0.43) | 0.16 (0.00-0.33) | 0.85 (0.84-0.86) | 0.18 (0.00-0.36) | 0.15 (0.00-0.31) | 0.11 (0.10-0.12) |
| NB | 0.78 (0.74-0.82) | 0.37 (0.32-0.41) | 0.61 (0.55-0.67) | 0.78 (0.75-0.80) | 0.46 (0.41-0.51) | 0.35 (0.29-0.41) | 0.21 (0.18-0.23) |
| SVM | 0.80 (0.73-0.86) | 0.70 (0.56-0.83) | 0.14 (0.11-0.18) | 0.86 (0.85-0.87) | 0.23 (0.18-0.29) | 0.27 (0.20-0.33) | 0.10 (0.10-0.11) |
| LR | 0.80 (0.74-0.87) | 0.39 (0.34-0.45) | 0.70 (0.61-0.79) | 0.78 (0.75-0.82) | 0.50 (0.44-0.57) | 0.41 (0.32-0.50) | 0.16 (0.15-0.18) |
| Test set |  |  |  |  |  |  |  |
| LightGBM | 0.97 | 0.83 | 0.69 | 0.93 | 0.75 | 0.72 | 0.05 |
| GBM | 0.85 | 1.00 | 0.03 | 0.86 | 0.06 | 0.16 | 0.11 |
| RF | 0.77 | 0.75 | 0.17 | 0.87 | 0.28 | 0.32 | 0.11 |
| KNN | 0.63 | 0.44 | 0.23 | 0.95 | 0.30 | 0.24 | 0.14 |
| MLP | 0.78 | 0.48 | 0.31 | 0.85 | 0.38 | 0.30 | 0.11 |
| NB | 0.78 | 0.36 | 0.57 | 0.79 | 0.44 | 0.33 | 0.20 |
| SVM | 0.83 | 0.88 | 0.20 | 0.88 | 0.33 | 0.38 | 0.10 |
| LR | 0.79 | 0.34 | 0.69 | 0.75 | 0.45 | 0.35 | 0.16 |

* ML: Machine learing; AUROC: Area under the receiver operating characteristic curve; MCC: Matthew’s correlation coefficient; BSL: Brier score loss; LightGBM: Light Gradient Boosting Machine; RF: Random Forest; KNN: K-Nearest Neighbors; MLP: Multi-Layer Perceptron; NB: Naive Bayes; SVM: Support Vector Machine; LR: Logistic Regression.

**Table S3.** Performance of eight ML models in predicting CKD by leveraging the all features*.

| Target | AUROC | Precision | Recall | Accuracy | F1 score | MCC | BSL |
| --- | --- | --- | --- | --- | --- | --- | --- |
| Validation set |  |  |  |  |  |  |  |
| LightGBM | 0.89 (0.87-0.91) | 0.60 (0.51-0.69) | 0.43 (0.35-0.51) | 0.90 (0.89-0.92) | 0.49 (0.41-0.56) | 0.45 (0.37-0.52) | 0.08 (0.07-0.09) |
| GBM | 0.81 (0.77-0.86) | 0.00 (0.00-0.00) | 0.00 (0.00-0.00) | 0.89 (0.89-0.89) | 0.00 (0.00-0.00) | 0.00 (0.00-0.00) | 0.09 (0.09-0.09) |
| RF | 0.75 (0.72-0.78) | 0.50 (0.27-0.73) | 0.12 (0.06-0.17) | 0.89 (0.88-0.90) | 0.18 (0.10-0.26) | 0.20 (0.10-0.30) | 0.09 (0.09-0.09) |
| KNN | 0.60 (0.55-0.65) | 0.35 (0.22-0.48) | 0.10 (0.06-0.14) | 0.88 (0.87-0.89) | 0.15 (0.09-0.21) | 0.14 (0.07-0.21) | 0.11 (0.10-0.12) |
| MLP | 0.83 (0.79-0.87) | 0.00 (0.00-0.00) | 0.00 (0.00-0.00) | 0.89 (0.89-0.89) | 0.00 (0.00-0.00) | 0.00 (0.00-0.00) | 0.08 (0.08-0.09) |
| NB | 0.80 (0.76-0.84) | 0.31 (0.27-0.35) | 0.62 (0.52-0.72) | 0.80 (0.77-0.83) | 0.41 (0.36-0.46) | 0.33 (0.27-0.40) | 0.18 (0.16-0.20) |
| SVM | 0.79 (0.74-0.85) | 0.68 (0.44-0.91) | 0.12 (0.06-0.18) | 0.90 (0.89-0.90) | 0.20 (0.11-0.29) | 0.25 (0.14-0.36) | 0.08 (0.08-0.09) |
| LR | 0.86 (0.84-0.87) | 0.25 (0.22-0.27) | 0.88 (0.83-0.93) | 0.68 (0.65 -0.72) | 0.38 (0.35-0.42) | 0.35 (0.30-0.39) | 0.23 (0.21-0.25) |
| Test set |  |  |  |  |  |  |  |
| LightGBM | 0.96 | 0.64 | 0.78 | 0.95 | 0.70 | 0.68 | 0.05 |
| GBM | 0.84 | 1.00 | 0.06 | 0.93 | 0.11 | 0.23 | 0.06 |
| RF | 0.83 | 0.71 | 0.28 | 0.94 | 0.04 | 0.42 | 0.06 |
| KNN | 0.68 | 0.14 | 0.11 | 0.88 | 0.13 | 0.06 | 0.09 |
| MLP | 0.86 | 0.00 | 0.00 | 0.92 | 0.00 | 0.00 | 0.07 |
| NB | 0.85 | 0.22 | 0.78 | 0.77 | 0.34 | 0.32 | 0.21 |
| SVM | 0.89 | 0.55 | 0.33 | 0.93 | 0.41 | 0.39 | 0.06 |
| LR | 0.89 | 0.16 | 0.89 | 0.64 | 0.28 | 0.28 | 0.26 |

* ML: Machine learing; AUROC: Area under the receiver operating characteristic curve; MCC: Matthew’s correlation coefficient; BSL: Brier score loss; LightGBM: Light Gradient Boosting Machine; RF: Random Forest; KNN: K-Nearest Neighbors; MLP: Multi-Layer Perceptron; NB: Naive Bayes; SVM: Support Vector Machine; LR: Logistic Regression.

**Table S4.** Performance of eight ML models in predicting AKD by utilizing the top ten features on the test set*.

| Model | AUROC | Precision | Recall | Accuracy | False positive rate | False negative rate | F1 score | MCC | BSL |
| --- | --- | --- | --- | --- | --- | --- | --- | --- | --- |
| LightGBM | 0.94 | 0.67 | 0.8 | 0.91 | 0.07 | 0.20 | 0.73 | 0.68 | 0.07 |
| GBM | 0.90 | 0.80 | 0.11 | 0.86 | 0.01 | 0.89 | 0.20 | 0.27 | 0.09 |
| RF | 0.89 | 0.76 | 0.54 | 0.91 | 0.03 | 0.46 | 0.63 | 0.59 | 0.07 |
| KNN | 0.76 | 0.64 | 0.46 | 0.88 | 0.05 | 0.54 | 0.53 | 0.48 | 0.10 |
| MLP | 0.85 | 0.76 | 0.46 | 0.9 | 0.03 | 0.54 | 0.57 | 0.54 | 0.09 |
| NB | 0.82 | 0.42 | 0.6 | 0.82 | 0.15 | 0.40 | 0.49 | 0.4 | 0.15 |
| SVM | 0.80 | 0.67 | 0.29 | 0.87 | 0.03 | 0.71 | 0.4 | 0.38 | 0.09 |
| LR | 0.80 | 0.32 | 0.66 | 0.74 | 0.24 | 0.34 | 0.43 | 0.32 | 0.17 |

* ML: Machine learing; AUROC: Area under the receiver operating characteristic curve; MCC: Matthew’s correlation coefficient; BSL: Brier score loss;LightGBM: Light Gradient Boosting Machine; RF: Random Forest; KNN: K-Nearest Neighbors; MLP: Multi-Layer Perceptron; NB: Naive Bayes ; SVM: Support Vector Machine; LR: Logistic Regression.

**Table S5.** Performance of eight ML models in predicting CKD by utilizing the top five features on the test set*.

| Model | AUROC | Precision | Recall | Accuracy | False positive rate | False negative rate | F1 score | MCC | BSL |
| --- | --- | --- | --- | --- | --- | --- | --- | --- | --- |
| LightGBM | 0.94 | 0.43 | 0.72 | 0.91 | 0.08 | 0.28 | 0.54 | 0.51 | 0.07 |
| GBM | 0.91 | 0.78 | 0.39 | 0.94 | 0.01 | 0.61 | 0.52 | 0.53 | 0.05 |
| RF | 0.88 | 0.60 | 0.67 | 0.94 | 0.04 | 0.33 | 0.63 | 0.60 | 0.05 |
| KNN | 0.77 | 0.53 | 0.50 | 0.93 | 0.04 | 0.50 | 0.51 | 0.48 | 0.07 |
| MLP | 0.93 | 0.69 | 0.50 | 0.94 | 0.02 | 0.50 | 0.58 | 0.56 | 0.05 |
| NB | 0.92 | 0.24 | 0.89 | 0.78 | 0.23 | 0.11 | 0.38 | 0.39 | 0.16 |
| SVM | 0.92 | 0.67 | 0.33 | 0.94 | 0.01 | 0.67 | 0.44 | 0.44 | 0.05 |
| LR | 0.92 | 0.19 | 0.94 | 0.68 | 0.34 | 0.06 | 0.31 | 0.33 | 0.23 |

*ML: Machine learing; AUROC: Area under the receiver operating characteristic curve; MCC: Matthew’s correlation coefficient; BSL: Brier score loss;LightGBM: Light Gradient Boosting Machine; RF: Random Forest; KNN: K-Nearest Neighbors; MLP: Multi-Layer Perceptron; NB: Naive Bayes ; SVM: Support Vector Machine; LR: Logistic Regression.
